# Mutations in the *Caenorhabditis elegans* U2AF Large Subunit UAF-1 Alter the Choice of a 3′ Splice Site *In Vivo*

**DOI:** 10.1371/journal.pgen.1000708

**Published:** 2009-11-06

**Authors:** Long Ma, H. Robert Horvitz

**Affiliations:** Howard Hughes Medical Institute, Department of Biology, Massachusetts Institute of Technology, Cambridge, Massachusetts, United States of America; Stanford University Medical Center, United States of America

## Abstract

The removal of introns from eukaryotic RNA transcripts requires the activities of five multi-component ribonucleoprotein complexes and numerous associated proteins. The lack of mutations affecting splicing factors essential for animal survival has limited the study of the *in vivo* regulation of splicing. From a screen for suppressors of the *Caenorhabditis elegans unc-93(e1500)* rubberband Unc phenotype, we identified mutations in genes that encode the *C. elegans* orthologs of two splicing factors, the U2AF large subunit (UAF-1) and SF1/BBP (SFA-1). The *uaf-1(n4588)* mutation resulted in temperature-sensitive lethality and caused the *unc-93* RNA transcript to be spliced using a cryptic 3′ splice site generated by the *unc-93(e1500)* missense mutation. The *sfa-1(n4562)* mutation did not cause the utilization of this cryptic 3′ splice site. We isolated four *uaf-1(n4588)* intragenic suppressors that restored the viability of *uaf-1* mutants at 25°C. These suppressors differentially affected the recognition of the cryptic 3′ splice site and implicated a small region of UAF-1 between the U2AF small subunit-interaction domain and the first RNA recognition motif in affecting the choice of 3′ splice site. We constructed a reporter for *unc-93* splicing and using site-directed mutagenesis found that the position of the cryptic splice site affects its recognition. We also identified nucleotides of the endogenous 3′ splice site important for recognition by wild-type UAF-1. Our genetic and molecular analyses suggested that the phenotypic suppression of the *unc-93(e1500)* Unc phenotype by *uaf-1(n4588)* and *sfa-1(n4562)* was likely caused by altered splicing of an unknown gene. Our observations provide *in vivo* evidence that UAF-1 can act in regulating 3′ splice-site choice and establish a system that can be used to investigate the *in vivo* regulation of RNA splicing in *C. elegans*.

## Author Summary

Eukaryotic genes contain intervening intronic sequences that must be removed from pre-mRNA transcripts by RNA splicing to generate functional messenger RNAs. While studying genes that encode and control a presumptive muscle potassium channel complex in the nematode *Caenorhabditis elegans*, we found that mutations in two splicing factors, the U2AF large subunit and SF1/BBP suppress the rubberband Unc phenotype caused by a rare missense mutation in the gene *unc-93*. Mutations affecting the U2AF large subunit caused the recognition of a cryptic 3′ splice site generated by the *unc-93* mutation, providing *in vivo* evidence that the U2AF large subunit can affect splice-site selection. By contrast, an SF1/BBP mutation that suppressed the rubberband Unc phenotype did not cause splicing using this cryptic 3′ splice site. Our genetic studies identified a region of the U2AF large subunit important for its effect on 3′ splice-site choice. Our mutagenesis analysis of *in vivo* transgene splicing identified a positional effect on weak 3′ splice site selection and nucleotides of the endogenous 3′ splice site important for recognition. The system we have defined should facilitate future *in vivo* analyses of pre–mRNA splicing.

## Introduction

Eukaryotic genes contain intervening introns that are spliced from transcribed pre-mRNAs to generate functional coding mRNAs [1],[2]. Alternative splicing results in distinct mRNAs that encode proteins with distinct functions, increases the proteome size and is believed to be important to the biological complexity of metazoans [1],[3],[4]. In *C. elegans*, mRNA transcripts of at least 13% of predicted genes are alternatively spliced [5]. In humans, most genes are alternatively spliced [6],[7]. A dramatic example of alternative splicing is provided by the *Drosophila* gene *Dscam* (Down syndrome cell adhesion molecule), which through alternative splicing could potentially generate over 30,000 isoforms [8], some of which have been shown to play important roles in immune responses [9] and neuronal arborization [10]–[12]. Mutations affecting the splicing process or splicing machinery cause numerous human diseases [13],[14].

Pre-mRNA splicing involves five small nuclear ribonucleoprotein particles (snRNPs) and numerous associated factors [1],[2],[15]. The U1 snRNP recognizes the 5′ splice donor site through base-pairing between the U1 snRNA and the 5′ splice site of the target intron [16]. The recognition of the 3′ splice acceptor site is achieved by SF1/BBP (splicing factor one/branch-point binding protein) and the large and small subunits of U2AF (U2 auxiliary factor) [17]–[23]. In mammals, SF1/BBP binds a weak consensus branch-point sequence, the U2AF large subunit binds a long polypyrimidine sequence and the U2AF small subunit binds the 3′ splice site YAG [19], [21], [24]–[26]. The yeast *Saccharomyces cerevisiae* lacks a U2AF small subunit and a polypyrimidine sequence in its introns, and the recognition of a 3′ splice site is achieved by binding of SF1/BBP to a highly conserved consensus branch-point sequence [17],[24],[27],[28]. In the nematode *Caenorhabditis elegans*, there is no consensus branch-point sequence or long polypyrimidine sequence, and the recognition of a 3′ splice site is achieved by the binding of the U2AF large and small subunits to a consensus UUUUCAGR sequence in which “AG” is the 3′ splice site [23],[29].

Splicing is also regulated by many Arginine-Serine-rich RNA-binding SR proteins [30]–[33] and hnRNP RNA-binding proteins [4]. These splicing factors recognize enhancer or silencer sequences in exons and introns to regulate the specificity and efficiency of splicing [4]. The genetic interactions among splicing factors and how signaling events regulate splicing efficiency and specificity are only partially understood.

*C. elegans* is a genetically tractable organism and has been used to study a broad variety of biological problems. Our laboratory has analyzed a set of genes, *unc-93*, *sup-9* and *sup-10*, that encode components of a presumptive *C. elegans* two-pore domain K^+^ channel complex and regulate muscle activity [34]–[37]. Rare gain-of-function (gf) mutations in any of these three genes cause abnormal body-muscle contraction and are thought to activate the SUP-9 K^+^ channel. The gf mutant animals are defective in egg laying, sluggish and exhibit a rubberband phenotype: when prodded on the head, the animal contracts and relaxes along its entire body without moving backwards. Complete loss-of-function (lf) mutations of *unc-93*, *sup-9* and *sup-10* do not cause any obvious abnormalities [35],[36]. The SUP-9 protein is similar to the mammalian Two-pore Acid Sensitive K ^+^ channels TASK-1 and TASK-3 [34]. *sup-10* encodes a novel single-transmembrane domain protein without identified mammalian homologs [34]. *unc-93* encodes a multiple transmembrane-domain protein that defines a novel family of proteins conserved from *C. elegans* to mammals [34],[37]. A mammalian UNC-93 homolog, UNC-93b, plays important roles in the innate immune response, probably by regulating signals mediated through Toll-like receptors [38]–[42].

Previous genetic screens for genes that affect the activities of *unc-93*, *sup-9* and *sup-10* genes were not designed to identify genes essential for fertility or animal survival. To seek such essential genes, we performed a clonal genetic screen for suppressors of the locomotion defect caused by the *unc-93* gf mutation *e1500*. In this paper, we describe our studies of two suppressors identified from this screen and the establishment of a reporter system for *in vivo* analysis of RNA splicing in *C. elegans*. We suggest that the U2AF large subunit affects 3′ splice site recognition and that some aspect of the function of the putative UNC-93/SUP-9/SUP-10 two-pore domain potassium channel complex depends on an unidentified gene the processing of which requires the functions of the U2AF large subunit and SF1/BBP.

## Results

### A clonal screen identified two new *unc-93(e1500)* suppressor genes essential for suvival

Rare gf mutations in the *C. elegans* genes *unc-93*, *sup-9* and *sup-10* cause a rubberband Unc phenotype, while lf mutations in these genes result in a phenotypically wild-type phenotype [35],[36],[43]. Previous screens for suppressors of the rubberband Unc phenotype were not designed to identify genes essential for animal survival [35],[36],[43],[44]. We performed a clonal genetic screen to seek new suppressors of the rubberband Unc phenotype caused by the *unc-93(e1500)* mutation, with the goal of identifying mutations that also cause sterility or lethality (see Materials and Methods). We screened about 10,000 F_1_ progeny (about 20,000 mutagenized haploid genomes) of P_0_ animals mutagenized with EMS (ethyl methanesulfonate) and isolated the suppressors *n4588* and *n4562*. *n4588* causes embryonic lethality at 25°C, and *n4562* causes sterility at all temperatures. By mapping these mutations, we found that *n4588* and *n4562* are not alleles of any previously characterized suppressors of the rubberband Unc phenotype (see Materials and Methods).

### *n4588* is a missense mutation in *uaf-1*, which encodes the *C. elegans* ortholog of the U2AF large subunit splicing factor

**Figure 1:**
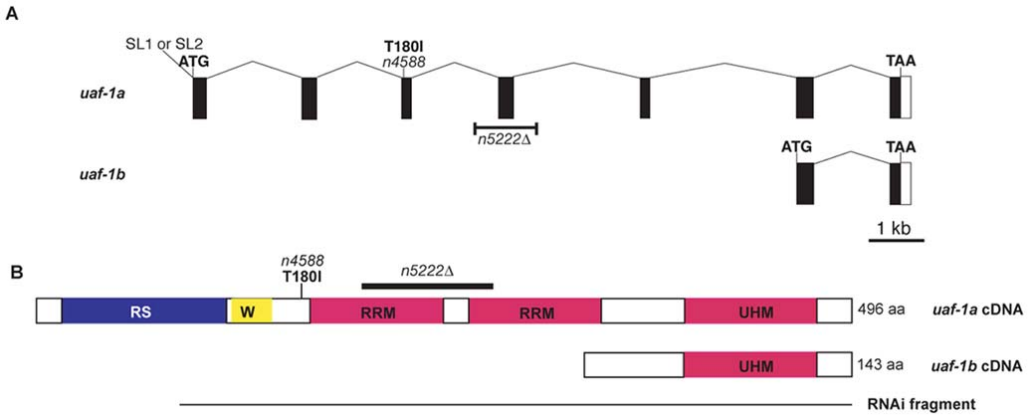
*uaf-1* gene and proteins. (A) Genomic structure of the *uaf-1* isoforms *uaf-1a* and *uaf-1b* (adapted from Wormbase WS189) [45]. The locations of the *n4588* missense mutation and the *n5222* deletion allele are indicated. Black boxes: coding exons. Open box: 3′ UTR. Positions of start (ATG) and stop codons (TAA) are indicated. SL1 and SL2, splice leaders associated with the *uaf-1a* transcript [45]. (B) Predicted UAF-1 protein domains encoded by *uaf-1a* and *uaf-1b* cDNAs, the position of the T180I change caused by the *n4588* mutation and the domains affected by the *n5222*Δ deletion are shown. RNAi fragment: the portion of the *uaf-1a* cDNA used within a dsRNA-expressing plasmid for RNAi. RS: Arginine-Serine rich domain. W: U2AF small subunit-interacting domain. RRM: RNA recognition motif. UHM: U2AF homology motif.

*n4588* is a strong recessive suppressor of the locomotion defect and rubberband phenotype of *unc-93(e1500)* animals (Table 1). *n4588* is or is closely linked to a mutation that causes a recessive temperature-sensitive (ts) lethal phenotype and results in embryonic lethality at 25°C (see Materials and Methods) (data not shown). At 20°C the lethal phenotype was incompletely penetrant. At 15°C *n4588* animals appeared similar to wild-type animals. We mapped the ts-lethal phenotype of *n4588* animals to an 80 kb region on the left arm of LG III (see Materials and Methods). By determining the sequences of the coding exons of four of eight genes located within this 80 kb interval, we found a point mutation in the third coding exon of the major isoform of the gene *uaf-1* (*uaf-1a* in Figure 1A), changing codon 180 from ACT to ATT, a change predicted to replace a conserved threonine with an isoleucine. *uaf-1a* encodes the *C. elegans* ortholog of the highly conserved U2AF large subunit (U2AF, U2 auxiliary factor) [45]. In mammals, the U2AF large subunit binds a polypyrimidine sequence preceding the 3′ splice site [25],[46] to regulate pre-mRNA splicing. In *C. elegans*, together with the U2AF small subunit ortholog UAF-2, UAF-1 binds a consensus UUUUC**AG**R sequence, in which AG is the 3′ splice site [23],[29]. The U2AF large subunit contains an RS-rich (Arginine-Serine) domain (Figure 1B, RS), a U2AF small subunit-interacting domain (Figure 1B, W), two RRM (RNA recognition motif) domains (Figure 1B, RRM) [26],[47] and a C-terminal UHM (U2AF homology motif) domain that binds the splicing factor SF1/BBP [48]. The T180I change caused by the *n4588* mutation lies between the U2AF small subunit-interacting domain and the first RRM domain of UAF-1a (Figure 1B).

**Table 1:** Suppression of *unc-93(e1500)* and *sup-10(n983)* by *uaf-1* and *sfa-1* mutations.

Genotype	Bodybends/30 sec±SD	Rubberband phenotype	n
*wild-type*	20.4±3.7	None	15
*uaf-1(n4588)*	23.1±4.1	None	20
*sfa-1(n4562)*	26.5±4.9	None	20
*unc-93(e1500)*	0.9±1.2	Strong	15
*uaf-1(n4588) unc-93(e1500)*	21.0±5.5	None	20
*uaf-1(n4588) unc-93(e1500)/uaf-1*(*n5222*Δ) *unc-93(e1500)*	22.2±2.4	None	20
*unc-93(e1500)*; *sfa-1(n4562)*	13.5±4.9	Weak	15
*unc-93(n200)*	15.6±3.5	Weak	20
*uaf-1(n4588) unc-93(n200)*	10.2±2.2	Weak	20
*unc-93(n200)*; *sfa-1(n4562)*	13.3±2.5	Weak	20
*sup-10(n983)*	4.3±1.9	Moderate	15
*uaf-1(n4588)*; *sup-10(n983)*	24.5±4.1	None	20
*sfa-1(n4562)*; *sup-10(n983)*	18.8±4.0	Weak	15
*sup-9(n1550)*; *sup-18(n1014)* *	0.1±0.4	Severe	15
*sup-9(n1550)*; *uaf-1(n4588) sup-18(n1014)* *	0.1±0.2	Severe	20
*sup-9(n1550)*; *sup-18(n1014)* *; *sfa-1(n4562)*	1.0±1.3	Severe	12

Locomotion and the rubberband phenotype were scored as described in Materials and Methods. Genotypes were as listed.

*: *sup-18(n1014)* was included to allow the survival of *sup-9(n1550)* animals. SD: Standard deviation.

### Expression of *uaf-1a* in body-wall muscles rescued the suppression of *unc-93(e1500)* by *n4588*

To test whether the point mutation found in the *uaf-1a* isoform caused the suppressor activity of *n4588*, we generated transgenic animals expressing a UAF-1a::GFP fusion protein under the control of a *myo-3* myosin promoter, which drives transgene expression in body-wall muscle cells [49]. This *uaf-1a* cDNA, which encodes a predicted full-length UAF-1 protein, restored the Unc phenotype when expressed in *uaf-1(n4588) unc-93(e1500)* animals (Figure 1B and Table 2). A predicted short *uaf-1* isoform, *uaf-1b*, which contains only part of the second RRM domain and the C-terminal UHM domain, failed to restore the Unc phenotype (Figure 1B and Table 2). Expression of these *myo-3*-driven transgenes (*uaf-1a* and *uaf-1b*) in wild-type animals did not cause a rubberband Unc phenotype or any other visible abnormality (data not shown). Introducing stop codons or the *n4588* T180I mutation into the full-length *uaf-1a* cDNA abrogated its rescuing activity (Table 2). Heat-shock-driven expression of a transgene expressing the full-length *uaf-1a* cDNA under control of a heat-shock promoter [50] partially rescued both the suppression of *unc-93(e1500)* by *uaf-1(n4588)* and the ts-lethality caused by *uaf-1(n4588)* (Table 2 and data not shown), suggesting that the T180I mutation also caused the ts-lethal phenotype. Feeding *unc-93(e1500)* animals with *uaf-1* RNAi-expressing bacteria (Figure 1B and Materials and Methods) also partially suppressed the Unc phenotype (Table 2), suggesting that normal expression of *uaf-1* is required for the rubberband Unc phenotype caused by *unc-93(e1500)*.

**Table 2:** *uaf-1a* and *sfa-1* transgenes rescued the suppression of the rubberband Unc phenotype of *unc-93(e1500)* animals by *uaf-1(n4588)* and *sfa-1(n4562)*.

Genotype	Phenotype	Rescued lines/Total lines
*unc-93(e1500)*	Strong Unc	NA
*uaf-1(n4588) unc-93(e1500)*	Non-Unc	NA
*uaf-1(n4588) unc-93(e1500)*; *nEx[P_myo-3_uaf-1a cDNA::gfp]*	Strong Unc	12/12
*uaf-1(n4588) unc-93(e1500)*; *nEx[P_myo-3_uaf-1b cDNA::gfp]*	Non-Unc	0/4
*uaf-1(n4588) unc-93(e1500)*; *nEx[P_myo-3_uaf-1a cDNA(S178Opal, Q182Ochre)::gfp]*	Non-Unc	0/7
*uaf-1(n4588) unc-93(e1500)*; *nEx[P_myo-3_uaf-1a cDNA(T180I)::gfp]*	Non-Unc	0/2
*uaf-1(n4588) unc-93(e1500)*; *nEx[P_hsp16-41_uaf-1a cDNA::gfp]*	Moderate Unc	5/13
*uaf-1(RNAi) unc-93(e1500)*	Weak Unc	NA
*unc-93(e1500)*; *sfa-1(n4562)*	Weak Unc	NA
*unc-93(e1500)*; *sfa-1(n4562)*; *nEx[P_myo-3_sfa-1 cDNA::gfp]*	Strong Unc	6/6
*unc-93(e1500)*; *sfa-1(RNAi)*	Weak Unc	NA

A wild-type *uaf-1a* cDNA expressed in body-wall muscles rescued the suppression of *unc-93(e1500)* by *uaf-1(n4588)*. A cDNA for the shorter *uaf-1* isoform, *uaf-1b*, failed to rescue. Introducing stop codons or the *n4588* T180I mutation into the *uaf-1a* cDNA abrogated the rescuing activity. Inducing *uaf-1* expresssion with heat-shock partially rescued the suppression of *unc-93(e1500)*. Reducing *uaf-1* expression by RNAi partially suppressed the rubberband Unc phenotype of *unc-93(e1500)*. A wild-type *sfa-1* cDNA expressed in body-wall muscles of *unc-93(e1500)*; *sfa-1(n4562)* animals restored the rubberband Unc phenotype. Reducing *sfa-1* expression by RNAi partially suppressed the rubberband Unc phenotype. Total lines: number of stable independent transgenic lines that expressed the transgenes. Rescued lines: number of transgenic lines that the rubberband Unc phenotype of *unc-93(e1500)* was restored. Non-transgenic animals were used for comparison. NA: Not applicable.

We isolated a *uaf-1* deletion mutation, *n5222*Δ, which removes the fourth exon (encoding part of the first RRM and part of the second RRM of UAF-1a) of the *uaf-1a* isoform (Figure 1A) and is predicted to cause a frameshift after amino acid 229 if the third and fifth exons of the *uaf-1a* isoform are spliced together. *uaf-1*(*n5222*Δ)/+ animals grew and moved like the wild type, and *uaf-1*(*n5222*Δ)/+ did not suppress the rubberband Unc phenotype of *unc-93(e1500)* animals (data not shown). *uaf-1*(*n5222*Δ) homozygous mutants arrested and died at the late L1 to early L2 larval stages (based on body size), which precluded examination of the rubberband Unc behavior of *n5222*Δ homozygous animals (see Materials and Methods). *uaf-1(n4588)/uaf-1*(*n5222*Δ) suppressed the rubberband Unc phenotype of *unc-93(e1500)* animals as strongly as did homozygous *uaf-1(n4588)* (Table 1). Similar to *uaf-1(n4588)* homozygotes, *uaf-1(n4588)/uaf-1*(*n5222*Δ) animals died embryonically at 25°C (data not shown). These results establish that *n4588* is an allele of *uaf-1* and that reducing the dosage of the *uaf-1(n4588)* allele by 50% does not affect the suppression of the rubberband Unc phenotype of *unc-93(e1500)* animals. These data suggest that *uaf-1(n4588)* causes either a reduction/loss of *uaf-1* activity or an altered *uaf-1* activity that is antagonized by the wild-type *uaf-1* gene (see Discussion).

### *uaf-1(n4588)* differentially suppressed different rubberband Unc mutants

Locomotion defects similar to those caused by *unc-93(e1500)* are also caused by the *unc-93(n200)* mutation [36] and by gf mutations in the genes *sup-9* and *sup-10* [35],[43]. We tested whether the *uaf-1(n4588)* mutation could suppress the Unc phenotype caused by these other mutations (Table 1). Neither the weak locomotion defect nor the weak rubberband Unc defect caused by *unc-93(n200)* was suppressed by *uaf-1(n4588)* (Table 1). *sup-10(n983)*, which causes a rubberband Unc phenotype that is more severe than that of *unc-93(n200)* animals but less severe than that of *unc-93(e1500)* animals, was completely suppressed by *uaf-1(n4588)* (Table 1). The strongest rubberband mutant, *sup-9(n1550)* [43], was not suppressed by *uaf-1(n4588)* (Table 1). These data suggest that *uaf-1(n4588)* is an allele-specific suppressor of *unc-93* but not a gene-specific suppressor of the rubberband Unc mutants and is distinct in its suppression pattern from other known suppressors of *unc-93*, *sup-9* and *sup-10* (see Discussion).

Null mutations of *unc-93*, *sup-10* and *sup-9* do not cause visible abnormalities in a wild-type background [35],[36]. We tested whether these genes might function redundantly with *uaf-1*, by generating double mutants containing *uaf-1(n4588)* and null mutations of *unc-93*, *sup-10* or *sup-9*. We found that such double mutant animals grew and behaved indistinguishably from *uaf-1(n4588)* single mutant animals (Table S1), suggesting that *unc-93*, *sup-10* and *sup-9* are not functionally redundant with *uaf-1*. To examine whether *uaf-1(n4588)* can suppress gf mutations affecting other two-pore domain potassium channels, we generated double mutant animals containing the *uaf-1(n4588)* mutation and the *unc-58(e665sd)* [51] (J. Thomas, personal communication), *egl-23(n601sd)* [52] (J. Thomas, personal communication) or *twk-18(e1913sd)* [53] mutations. The behavioral defects of these mutants were not suppressed by *uaf-1(n4588)* (Table S1).

To determine if the expression of *unc-93*, *sup-9*, *sup-10* or any of the other genes known to interact with these genes is reduced in *uaf-1(n4588)* animals, we examined *unc-93*, *sup-10*, *sup-9*, *sup-18* [35] (I. Perez de la Cruz and H.R.H., unpublished results) and *sup-11* [54] (E. Alvarez-Saavedra and H.R.H, unpublished results) mRNA levels. Like lf mutations in *unc-93*, *sup-10* and *sup-9*, lf mutations in *sup-18* and gf mutations in *sup-11* can suppress the rubberband Unc phenotype caused by gf mutations in *unc-93*, *sup-9* and *sup-10*. Using real-time qRT–PCR, we found no obvious reduction of the mRNA levels of these five genes (Figure S1). We also examined the expression of UAF-1 protein using western blotting [45] and found no apparent difference in UAF-1 protein levels between wild-type and *uaf-1(n4588)* animals (data not shown), suggesting that the suppression of *unc-93(e1500)* by *uaf-1(n4588)* is not caused by a reduction of the level of the UAF-1 protein.

### *uaf-1(n4588)* altered the splicing of *unc-93(e1500)* exon 9 by recognizing a cryptic 3′ splice site generated by the *unc-93(e1500)* missense mutation

**Figure 2:**
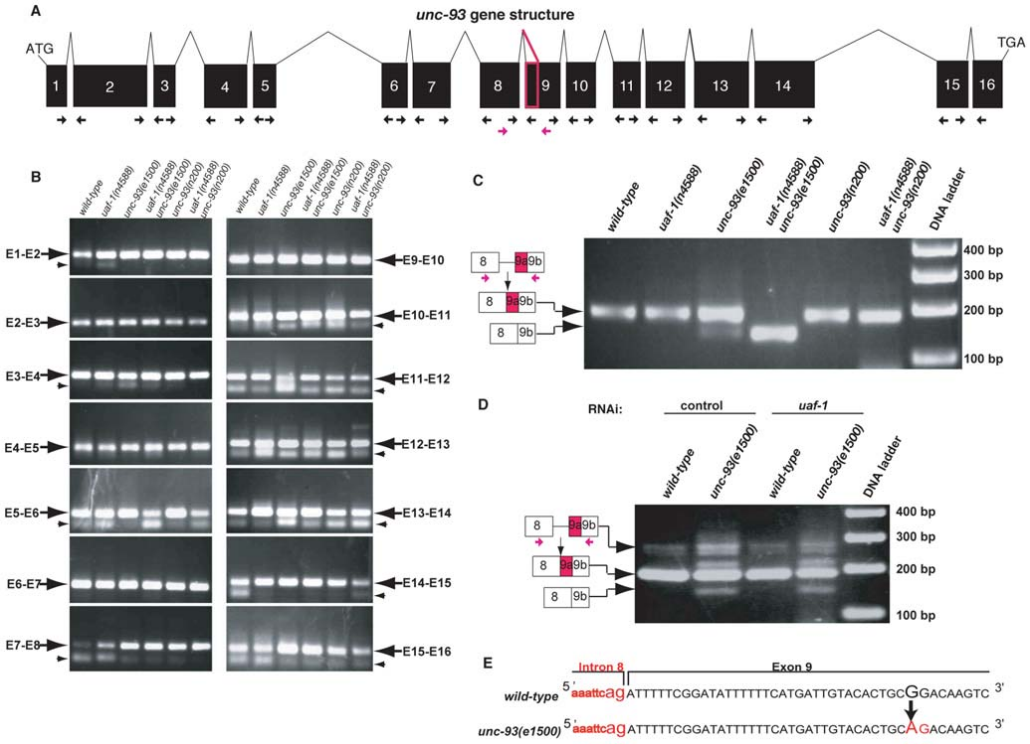
*uaf-1(n4588)* dramatically alters *unc-93(e1500)* but not *unc-93(+)* exon 9 splicing. (A) Genomic structure of the *unc-93* gene. Exons of *unc-93* are indicated by black boxes and introns by thin lines. The part of exon 9 that was removed by alternative splicing in *uaf-1(n4588) unc-93(e1500)* animals is marked as a red box. Exonic primers (arrows) flanking each intron were used to amplify mRNA regions of each exon-exon junction. The red primer pair was used to identify the alternative splice products of *unc-93(e1500)* exon 9. (B) RT–PCR experiments to examine the splicing of *unc-93* mRNA. En-En+1 (arrows) indicates the junction of two adjacent exons. Genotypes of each sample are indicated at the top. Small arrowheads indicate primer dimers, which form variably in RT–PCR experiments and are template-independent. (C) A weak alternatively spliced *unc-93* exon 9 was detected in *unc-93(e1500)* animals (lane 3, lower arrow). In *uaf-1(n4588) unc-93(e1500)* animals (lane 4, lower arrow), this alternative spliced product was dramatically enhanced. The diagrams on the left illustrate the splicing events responsible for the generation of each band. The upper band reflects the splicing seen for wild-type *unc-93*, while the lower band reflects the use of a cryptic 3′ splice site generated by the *unc-93(e1500)* missense mutation. Red arrows indicate the positions of PCR primers. Genotypes of each sample are listed at the top. (D) Reducing UAF-1 levels with RNAi did not cause increased splicing at the cryptic 3′ splice site found in *unc-93(e1500)* exon 9. (E) Partial genomic sequences of *unc-93* intron 8 (lowercase letters) and exon 9 (uppercase letters) in the wild type (above) and in *unc-93(e1500)* mutants (below). The G-to-A nucleotide change of the *e1500* mutation is indicated with an arrow. The AG sequence (red) forms a cryptic 3′ splice site that is recognized by the splicing machinery in *uaf-1(n4588)* animals.

We tested whether the splicing of *unc-93* is altered by *uaf-1(n4588)*. We examined the splicing of each exon of *unc-93* in wild-type, *uaf-1(n4588)*, *unc-93(e1500)*, *uaf-1(n4588) unc-93(e1500)*, *unc-93(n200)* and *uaf-1(n4588) unc-93(n200)* animals by RT–PCR (Figure 2). Every exon other than exon 9 of the *unc-93* gene was spliced similarly in all genotypes examined (Figure 2A and 2B). However, we had difficulty in consistently amplifying a cDNA band from *uaf-1(n4588) unc-93(e1500)* animals (data not shown) using the PCR primer pairs at the 3′ end of exon 8 and the 5′ end of exon 9 (indicated in black in Figure 2A). We therefore used a new pair of PCR primers that should amplify a larger region between exons 8 and 9 (Figure 2A, red arrows). With the new pair of PCR primers, we found that in *unc-93(e1500)* animals the region between exon 8 and 9 corresponded to a weak but consistent RT–PCR product of a reduced length (Figure 2C, lane 3, lower arrow), and this RT–PCR product was seen only in samples from *unc-93(e1500)* mutant animals (Figure 2C, lower arrow). In *uaf-1(n4588) unc-93(e1500)* animals, the RT–PCR product of reduced length was the most prominent product (Figure 2C, lane 4, lower arrow). We determined the sequence of this RT–PCR product and found that it was a consequence of an alternative splicing event that utilized a cryptic 3′ splice site in exon 9. This cryptic 3′ splice site was generated by the *unc-93(e1500)* missense mutation, which has a G-to-A transition that changes amino acid 388 from Gly to Arg [37] (Figure 2E). Quantification using Taqman RT–PCR (see Figure 4A for probe designs) indicated that the alternatively spliced exon 9 was about 1.3% of all spliced *unc-93* exon 9 in *unc-93(e1500)* animals and 68% in *uaf-1(n4588) unc-93(e1500)* animals (Figure 4B). Both non-quantitative (Figure 2C) and quantitative RT–PCR (Figure 4B) analyses failed to detect alternatively spliced exon 9 from wild-type, *uaf-1(n4588)*, *unc-93(n200)* or *uaf-1(n4588) unc-93(n200)* animals, all of which lack the cryptic 3′ splice site caused by the *unc-93(e1500)* mutation.

The alternatively spliced *unc-93* transcript is predicted to encode a truncated protein lacking 12 amino acids in one of the predicted transmembrane domains [37] (data not shown). To test whether the alternatively spliced *unc-93* transcript in *uaf-1(n4588) unc-93(e1500)* animals encoded a functional UNC-93 protein, we expressed the cDNA in the body-wall muscles of *sup-9(n1550)*; *unc-93*(*lr12*Δ) animals [34],[44] and found that this transgene did not restore the rubberband Unc phenotype (Table S2). By contrast, expression of the wild-type *unc-93* cDNA in these animals restored the severe rubberband Unc phenotype. These results suggested that the alternatively spliced *unc-93* transcript encoded a lf UNC-93 protein or possibly a dominant-negative UNC-93 protein. To test the latter possibility, we expressed either *unc-93* wild-type cDNA or the alternatively spliced *unc-93* cDNA in the body-wall muscles of *unc-93(e1500)* animals (Table S3). Consistent with previous observations that *unc-93(e1500)/+* animals have better locomotion than *unc-93(e1500)* animals [36],[37], overexpression of wild-type *unc-93* cDNA dramatically improved the locomotion of *unc-93(e1500)* animals (Table S3). If the alternatively spliced *unc-93* transcript encoded an UNC-93 protein that could interfere with the endogenous UNC-93 function and cause the suppression of the rubberband Unc phenotype by *uaf-1(n4588)* (68% alternatively spliced *unc-93* transcript), the transgene should also suppress the Unc phenotype of *unc-93(e1500)* animals. However, expression of the alternatively spliced *unc-93* transcript in the body-wall muscles did not suppress the Unc phenotype of *unc-93(e1500)* animals (Table S3), suggesting that the alternatively spliced *unc-93* transcript caused a loss of *unc-93* function and did not interfere with endogenous *unc-93* function.

To examine whether reducing UAF-1 expression, like the *uaf-1(n4588)* mutation, would alter the splicing of *unc-93(e1500)* exon 9, we fed animals with bacteria expressing dsRNA targeting *uaf-1* and assessed *unc-93* exon 9 splicing. As shown in Figure 2D and Figure 4B, reducing UAF-1 did not increase the relative level of alternatively spliced *unc-93(e1500)* exon 9. The RNAi treatment did significantly reduce the level of UAF-1 protein (Figure S2). That reducing *uaf-1* expression with RNAi did not cause altered splicing of *unc-93(e1500)* exon 9 similarly to that by the *uaf-1(n4588)* mutation is consistent with the hypothesis that *uaf-1(n4588)* does not reduce the function of UAF-1a but rather alters the function of UAF-1a, which leads to the recognition of the cryptic 3′ splice site of *unc-93(e1500)* exon 9 (see Discussion). However, it is possible that *uaf-1(n4588)* reduces *uaf-1* function and that *uaf-1(RNAi)* does not reduce *uaf-1* function as much.

*uaf-1(n4588)* suppressed the rubberband Unc phenotype of *sup-10(n983)* animals but did not suppress the rubberband Unc phenotype of *unc-93(n200)* and *sup-9(n1550)* animals (Table 1). Quantitative RT–PCR did not indicate reduction of *sup-10* mRNA in *uaf-1(n4588)* animals (Figure S1). We examined whether the *sup-10(n983)* transcript was alternatively spliced in *uaf-1(n4588)* mutants. *uaf-1(n4588)* did not cause the appearance of a *sup-10* cDNA band different in size from the full-length *sup-10* cDNA (Figure S3A). We determined the sequences of the *sup-10* cDNA RT–PCR products from wild-type, *sup-10(n983)*, *uaf-1(n4588)* and *uaf-1(n4588)*; *sup-10(n983)* animals and failed to identify an alternatively spliced *sup-10* transcript (data not shown).

To test whether *uaf-1(n4588)* can affect the splicing of all genes known to be alternatively spliced, we tested for genetic interactions between *uaf-1(n4588)* and *unc-52(e669)*. *unc-52* encodes the *C. elegans* ortholog of human basement membrane-specific heparan sulfate proteoglycan core protein, and mutations affecting *unc-52* cause adult paralysis [55],[56]. The Unc phenotype of *unc-52(e669)* can be suppressed by lf mutations of *smu-1* and *smu-2*, genes that encode *C. elegans* homologs of mammalian splicing factors [57]–[59]. The *unc-52(e669)* mutation causes a pre-mature stop in *unc-52* exon 17 [60], and *smu-1* and *smu-2* lf mutations suppress *unc-52(e669)* by removing exon 17 and generating an alternatively spliced and functional transcript [58]. The *unc-52(e444)* mutation causes a pre-mature stop in *unc-52* exon 18, which is not removed in *smu-1* and *smu-2* mutant animals, leading to a transcript with a premature stop codon. Double mutants containing the *unc-52(e444)* mutation and the *smu-1* or *smu-2* mutations display an Unc phenotype [58]. We examined the Unc phenotypes of *unc-52(e669)*; *uaf-1(n4588)* and *unc-52(e444)*; *uaf-1(n4588)* animals and found that *uaf-1(n4588)* did not suppress either *unc-52(e669)* or *unc-52(e444)* (Table S1), implying that the *uaf-1(n4588)* mutation did not affect the alternative splicing of *unc-52(e669)* exon 17 and thus does not affect all cases of alternative splicing non-specifically.

### *n4562* is a nonsense mutation in *sfa-1*, which encodes the *C. elegans* ortholog of the splicing factor SF1/BBP

The mutation *n4562* was also isolated from our clonal screen as a suppressor of the rubberband Unc phenotype of *unc-93(e1500)* animals. The suppressed phenotype was recessive, and *n4562* caused a completely penetrant recessive sterility that was temperature independent and was tightly linked to its suppressor activity (see Materials and Methods). Like *uaf-1(n4588)*, *n4562* suppressed *unc-93(e1500)* and *sup-10(n983)* but did not suppress *unc-93(n200)* or *sup-9(n1550)* (Table 1). Therefore, *n4562* is also an allele-specific suppressor of *unc-93* gf mutations but not a gene-specific suppressor for the rubberband Unc genes.

**Figure 3:**
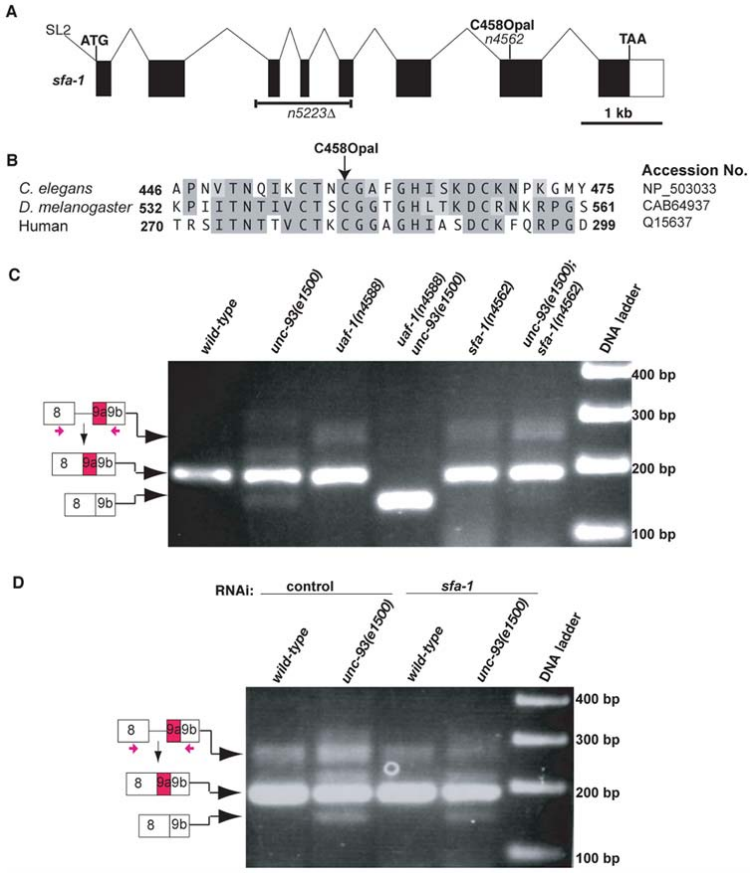
*sfa-1* gene and protein. (A) Predicted *sfa-1* gene structure (adapted from Wormbase WS189) [62]. The locations of the *n4562* nonsense mutation and the *n5223* deletion allele are indicated. SL2: splice leader associated with the *sfa-1* transcript [62]. (B) Partial sequence alignment of the conserved zinc finger domains of SF1/BBP orthologs from *C. elegans*, *D. melanogaster* and human. The amino acid numbers, the accession numbers and the Cys458Opal mutation are indicated. (C) Total RNAs were prepared from animals with the indicated genotypes, and RT–PCR experiments were performed to detect the splicing of *unc-93* exon 9. *sfa-1(n4562)* did not increase the alternative splicing of *unc-93(e1500)* exon 9. Genotypes are listed at the top. (D) Total RNAs were prepared from wild-type or *unc-93(e1500)* animals treated with control or *sfa-1* RNAi, and RT–PCR experiments were performed to detect the splicing of *unc-93* exon 9. Reducing SFA-1 by RNAi did not increase the alternative splicing of *unc-93(e1500)* exon 9. RNAi treatments are listed at the top.

We mapped *n4562* to the right of LG IV (see Materials and Methods). No known suppressors of *unc-93(e1500)* are located in this region. The genes *uaf-2*, encoding the *C. elegans* U2AF small subunit ortholog [61] and *Y116A8C.32*, encoding the SF1/BBP (splicing factor 1/branch-point binding protein) ortholog [62], are located in this genomic region and are expressed from the same operon together with three other genes (Wormbase WS189) [61],[62]. Orthologs of UAF-2 and SF1/BBP function with the ortholog of UAF-1 to regulate pre-mRNA splicing [2], leading us to consider these two genes as candidates for being mutated by *n4562*. We determined the DNA sequences of coding regions of *uaf-2* and *Y116A8C.32* from *n4562* animals and identified a nonsense mutation in *Y116A8C.32*, which we named *sfa-1* (*sfa*, splicing factor) (Figure 3A). *n4562* changed amino acid 458 from a Cys (TGT) to an opal stop (TGA) codon in a conserved C2HC-type zinc finger domain of the predicted SFA-1 protein (Figure 3A and 3B). This mutation is predicted to cause the expression of a truncated SFA-1 protein. We rescued the suppression of *unc-93(e1500)* by *sfa-1(n4562)* by expressing in body-wall muscles an SFA-1::GFP fusion protein driven by the *myo-3* promoter [49] (Table 2). Feeding *unc-93(e1500)* animals with bacteria expressing dsRNA targeting *sfa-1* partially suppressed the rubberband Unc phenotype (Table 2).

We isolated an *sfa-1* deletion mutation, *n5223*Δ, which removes the third and fourth exons and a majority of the fifth exon (Figure 3A). Together these regions are predicted to encode most (101 aa) of the U2AF large subunit-interacting domain (118 aa) of SFA-1 [62]. *n5223*Δ is predicted to cause a frameshift after amino acid 188 if the second exon and the residual fifth exon are spliced together. *sfa-1*(*n5223*Δ) caused recessive embryonic lethality, and *sfa-1*(*n5223*Δ)/*+* did not suppress the rubberband Unc phenotype of *unc-93(e1500)* animals (data not shown). *sfa-1(n4562)/*(*n5223*Δ) similarly caused embryonic lethalilty (data not shown), suggesting that the lethal phenotype of *sfa-1*(*n5223*Δ) homozygotes is caused by the *sfa-1*(*n5223*Δ) mutation. The embryonic lethality caused by *sfa-1*(*n5223*Δ) and *sfa-1(n4562)/sfa-1*(*n5223*Δ) precluded the use of *n5223*Δ for an analysis of the rubberband Unc phenotype, because our behavioral assay is performed with young adults (see Materials and Methods).

### *sfa-1(n4562)* did not cause alternative splicing of *unc-93(e1500)* exon 9 at the cryptic 3′ splice site

**Figure 4:**
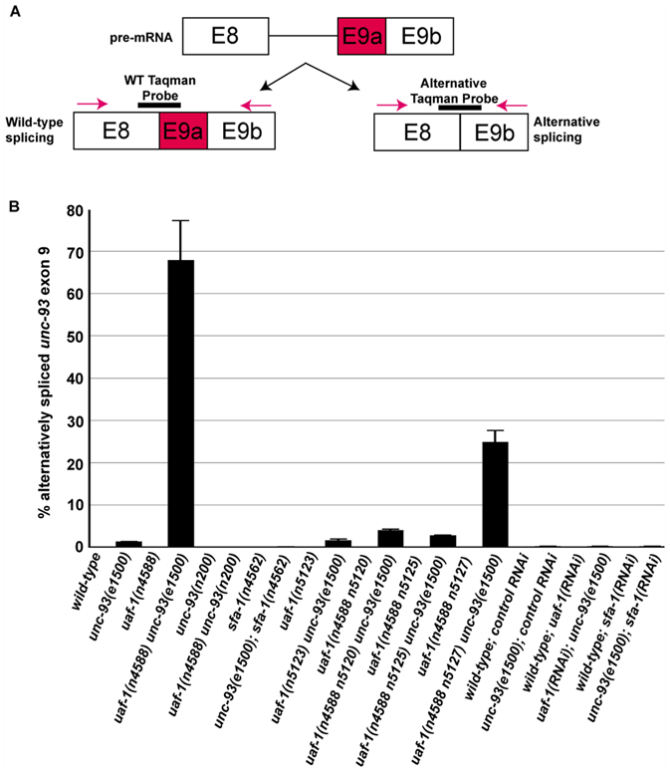
Taqman Real-time PCR quantification of *unc-93* alternative splicing. (A) Location of PCR primers (red arrows) and Taqman probes (dark short lines) for the quantification of *unc-93* exon 9 splicing. (B) Proportion of alternatively spliced exon 9 expressed as a percentage of total spliced (wild-type and alternative splice products) *unc-93* exon 9 in animals of different genotypes and animals treated with RNAi targeting the indicated genes. For every genotype except the RNAi-treated samples, each data set represents the average value of duplicate measurements of two biological replicates. For the RNAi-treated samples, each data set represents the average value of duplicate measurements of one biological sample. Error bars: standard errors of two biological replicates.

To test whether, like *uaf-1(n4588)*, *sfa-1(n4562)* caused alternative splicing of *unc-93(e1500)* exon 9, we used RT–PCR to examine the splicing of *unc-93(e1500)* exon 9. As shown in Figure 3C and Figure 4B, *sfa-1(n4562)* did not cause increased alternative splicing of *unc-93(e1500)* exon 9. We tested the effect of *sfa-1* on *unc-93(e1500)* exon 9 splicing by reducing *sfa-1* expression using RNAi (Figure 3D). *sfa-1(RNAi)* did not increase exon 9 alternative splicing (Figure 3D and Figure 4B).

Because (1) the *sfa-1(n4562)* mutation causes a recessive sterile phenotype, which is less severe than the recessive embryonic lethality caused by *sfa-1*(*n5223*Δ) (likely a null allele) or by *sfa-1(n4562)/sfa-1*(*n5223*Δ), (2) *sfa-1(RNAi)* phenocopies *sfa-1(n4562)* in the suppression of the rubberband Unc phenotype of *unc-93(e1500)* animals, and (3) *sfa-1(RNAi)* phenocopies *sfa-1(n4562)* in affecting the splicing of *unc-93(e1500)* exon 9, we propose that *sfa-1(n4562)* is a partial lf allele of *sfa-1* and that the suppression of the rubberband Unc phenotype of *unc-93(e1500)* animals by *sfa-1(n4562)* is likely caused by reduced *sfa-1* function.

To examine whether *sfa-1(n4562)* affects the alternative splicing of *unc-93(e1500)* exon 9 caused by the *uaf-1(n4588)* mutation, we generated *uaf-1(n4588)*; *sfa-1(n4562)* animals with or without *unc-93(e1500)*. Most *uaf-1(n4588)*; *sfa-1(n4562)* animals (with or without *unc-93(e1500)*) died embryonically, and the few that hatched arrested at the L2 larval stage (based on body size) (Figure S4). We failed to obtain a sufficient number of animals for RT–PCR analysis.

We also examined *sup-10* splicing in *sfa-1(n4562)* animals and failed to detect alternative splicing of the *sup-10* transcript (Figure S3B).

### Intragenic suppressors of *uaf-1(n4588)* ts-lethality differentially affected the alternative splicing of *unc-93(e1500)* exon 9

**Figure 5:**
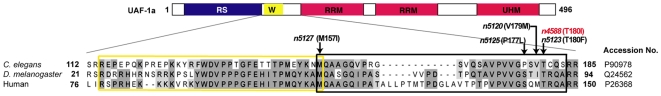
*uaf-1* mutations define a UAF-1 region that affects the recognition of different 3′ splice sites. Comparison of the sequences of the U2AF large subunit between the RS domain and the first RRM domain from *C. elegans*, *D. melanogaster* and human. The mutations caused by the *uaf-1(n4588)*, *uaf-1(n5120)*, *uaf-1(n5123)*, *uaf-1(n5125)* and *uaf-1(n5127)* mutations are indicated with arrows. RS: Arginine-Serine rich domain. W: U2AF small subunit-interacting domain. RRM: RNA recognition motif. UHM: U2AF homology motif. Black box: region of UAF-1a that might regulate the recognition of 3′ splice sites.

The ts-lethality of *uaf-1(n4588)* offered a genetic approach to seek new regulators of RNA splicing by screening for suppressors of the ts-lethal phenotype. We performed a genetic screen for suppressors of *uaf-1(n4588)* ts-lethality at 25°C. From this screen, we isolated four intragenic suppressors, *n5120*, *n5123*, *n5125* and *n5127* (Table 3) and seven extragenic suppressors (see Materials and Methods). To date we have characterized only the intragenic suppressors. *uaf-1(n5123)* caused an I180F (ATT-to-TTT) change at the same residue mutated by *n4588* (T180I) (ACT-to-ATT) (Table 3 and Figure 5) and eliminated the suppression of the locomotion defect of *unc-93(e1500)* (Table 4). *uaf-1(n4588 n5120)* is predicted to cause a V179M (GTG-to-ATG) change in addition to the *n4588* T180I mutation (Table 3 and Figure 5), and *uaf-1(n4588 n5120)* weakly suppressed *unc-93(e1500)* (Table 4). *uaf-1(n4588 n5125)* is predicted to cause a P177L (CCA-to-CTA) change in addition to the *n4588* T180I mutation (Table 3 and Figure 5), and *uaf-1(n4588 n5125)* also weakly suppressed *unc-93(e1500)* (Table 4). *uaf-1(n4588 n5127)* is predicted to cause an M157I (ATG-to-ATA) change in addition to the *n4588* T180I mutation (Table 3 and Figure 5) and was still a strong suppressor of *unc-93(e1500)* (Table 4). That the *unc-93(e1500)* suppressor activities of *uaf-1(n4588 n5120)*, *uaf-1(n4588 n5125)* and *uaf-1(n4588 n5127)* were caused by *uaf-1* mutations was confirmed by the observation that transgenes expressing *uaf-1a* (Figure 1A) in the body-wall muscles rescued the *unc-93(e1500)* suppressor phenotype of these mutants (Table 4). We quantified the splicing of *unc-93(e1500)* exon 9 in animals containing these *uaf-1* mutations using Taqman RT–PCR. As shown in Figure 4B, these *uaf-1* mutations exhibited differential effects on the splicing of *unc-93(e1500)* exon 9. *uaf-1(n5123)* had no apparent effect (1.6% alternative splicing vs. 1.3% for *unc-93(e1500)* alone). *uaf-1(n4588 n5120)* and *uaf-1(n4588 n5125)* weakly (4% and 2.8%, respectively) and *uaf-1(n4588 n5127)* moderately (25%) increased the alternative splicing of *unc-93(e1500)* exon 9 (Figure 4B). None of these mutations affected this splicing event as much as did *uaf-1(n4588)* (68%) (Figure 4B).

**Table 3:** Intragenic suppressors of *uaf-1(n4588)*.

Alleles of *uaf-1*	Mutations in UAF-1	Viability at 25°C
*n4588*	T180I	Lethal
*n5123*	T180F	Viable
*n4588 n5120*	V179M, T180I	Viable
*n4588 n5125*	P177L, T180I	Viable
*n4588 n5127*	M157I, T180I	Viable

List of allele numbers and changes of amino acids caused by intragenic suppressors of *uaf-1(n4588)*.

**Table 4:** Intragenic suppressors of *uaf-1(n4588)* suppress *unc-93(e1500)* differently.

Genotype	Bodybends/30 sec±SD	Rubberband phenotype	n
*wild-type*	20.4±3.7	None	20
*unc-93(e1500)*	0.9±1.2	Strong	20
*uaf-1(n5123) unc-93(e1500)*	0.6±0.8	Strong	20
*uaf-1(n5123)*	19.1±3.1	None	20
*uaf-1(n4588 n5120) unc-93(e1500)*	4.9±2.0	Moderate	20
*uaf-1(n4588 n5120)*	21.6±2.8	None	20
*uaf-1(n4588 n5120) unc-93(e1500); Transgene #1*	1.3±1.8	Strong	20
*uaf-1(n4588 n5120) unc-93(e1500)*; *Transgene #2*	0.9±1.4	Strong	20
*uaf-1(n4588 n5120) unc-93(e1500)*; *Transgene #3*	0.3±0.6	Strong	20
*uaf-1(n4588 n5125) unc-93(e1500)*	5.4±2.1	Moderate	20
*uaf-1(n4588 n5125)*	19.5±3.3	None	20
*uaf-1(n4588 n5125) unc-93(e1500)*; *Transgene #1*	0.6±0.9	Strong	20
*uaf-1(n4588 n5125) unc-93(e1500)*; *Transgene #2*	1.3±1.6	Strong	20
*uaf-1(n4588 n5125) unc-93(e1500)*; *Transgene #3*	0.8±1.4	Strong	20
*uaf-1(n4588 n5127) unc-93(e1500)*	21.5±3.6	Weak	20
*uaf-1(n4588 n5127)*	22.1±4.0	None	20
*uaf-1(n4588 n5127) unc-93(e1500)*; *Transgene #1*	1.3±1.7	Strong	20
*uaf-1(n4588 n5127) unc-93(e1500)*; *Transgene #2*	0.7±1.4	Strong	20
*uaf-1(n4588 n5127) unc-93(e1500)*; *Transgene #3*	1.6±2.0	Strong	20

To confirm that the suppression of *unc-93(e1500)* was caused by mutations in *uaf-1*, transgenes that express a wild-type *uaf-1a* cDNA in the body-wall muscles (*nEx[P_myo-3_uaf-1a cDNA::gfp]*) were introduced into the strains of the genotypes indicated and independent stable transgenic lines were established and scored. For the *uaf-1(n4588 n5120) unc-93(e1500)*, *uaf-1(n4588 n5125) unc-93(e1500)* and *uaf-1(n4588 n5127) unc-93(e1500)* genotypes, three independent lines were scored. Locomotion and the rubberband phenotype were assessed as described in Materials and Methods. SD: Standard deviation.

To test whether *uaf-1(n4588)* could suppress the Unc phenotype of *unc-93(e1500)* independently of *unc-93* splicing, we generated transgenic animals that overexpressed in the body-wall muscles a full-length *unc-93* cDNA containing the *e1500* gf mutation. Since this cDNA generates a fully spliced form of *unc-93* mRNA, if alternative splicing of *unc-93(e1500)* accounted for the suppression of the rubberband Unc phenotype, overexpressed *unc-93(e1500)* cDNA would not generate an alternatively spliced isoform and the animals would be as Unc in an *uaf-1(n4588)* background as in a wild-type background. As shown in Table 5, in wild-type animals, overexpression of the *unc-93(e1500)* cDNA caused a strong rubberband Unc phenotype. The presence of the *uaf-1(n4588)* mutation reduced the severity of the rubberband Unc phenotype caused by the same transgenes. Similarly, overexpression of the *unc-93(e1500)* cDNA also caused a weaker rubberband Unc phenotype in *sfa-1(n4562)* animals than in wild-type animals (Table 5). This result implied that *uaf-1(n4588)* can suppress the *unc-93(e1500)* rubberband Unc phenotype through mechanism(s) other than by affecting the splicing of *unc-93*. This finding was consistent with our results showing that although *sfa-1(n4562)* suppressed the rubberband Unc phenotype of *unc-93(e1500)* animals, *sfa-1(n4562)* did not affect the alternative splicing of *unc-93(e1500)* exon 9, which also suggested that the suppression of *unc-93(e1500)* by *sfa-1(n4562)* was mediated by a mechanism other than by affecting the alternative splicing of *unc-93* (see Discussion).

**Table 5:** *uaf-1(n4588)* and *sfa-1(n4562)* partially suppress the rubberband Unc phenotype caused by overexpression of *unc-93(e1500)* cDNA in body-wall muscles.

Genotype	Bodybends/30 sec±SD	n
*uaf-1(n4588)*; *Transgene #1*	7.8±3.9	15
*wild-type*; *Transgene #1*	3.7±2.9	15
*uaf-1(n4588)*; *Transgene #2*	4.6±2.3	15
*wild-type*; *Transgene #2*	2.9±2.0	15
*sfa-1(n4562)*; *Transgene #3*	8.2±2.1	15
*wild-type* *; *Transgene #3*	4.8±3.1	15
*sfa-1(n4562)*; *Transgene #4*	10.5±3.0	15
*wild-type* *; *Transgene #4*	6.5±4.4	15

Transgenes that expressed an *unc-93* cDNA with the *e1500* missense mutation (*nEx[P_myo-3_unc-93(e1500) cDNA::gfp]*) in body-wall muscles were introduced into *uaf-1(n4588)* and *sfa-1(n4562)/nT1[qIs51]* animals, and four independent stable transgenic lines were established. Transgenes in the *uaf-1(n4588)* background were backcrossed into the wild-type background. Transgenic *sfa-1(n4562)* animals were obtained as progeny of transgenic *sfa-1(n4562)/nT1[qIs51]* animals. The locomotion of the transgenic animals was assayed as described in Materials and Methods. Comparison should be made between animals of either wild-type or mutant genotypes carrying the same transgenic extra-chromosomal array.

*: “wild-type” refers to *sfa-1(n4562)/nT1[qIs51]*, which exhibits a wild-type phenotype.

### Nucleotide substitutions at the intron 8 endogenous 3′ splice site and the exon 9 cryptic 3′ splice site alter the recognition of these two sites differently

**Figure 6:**
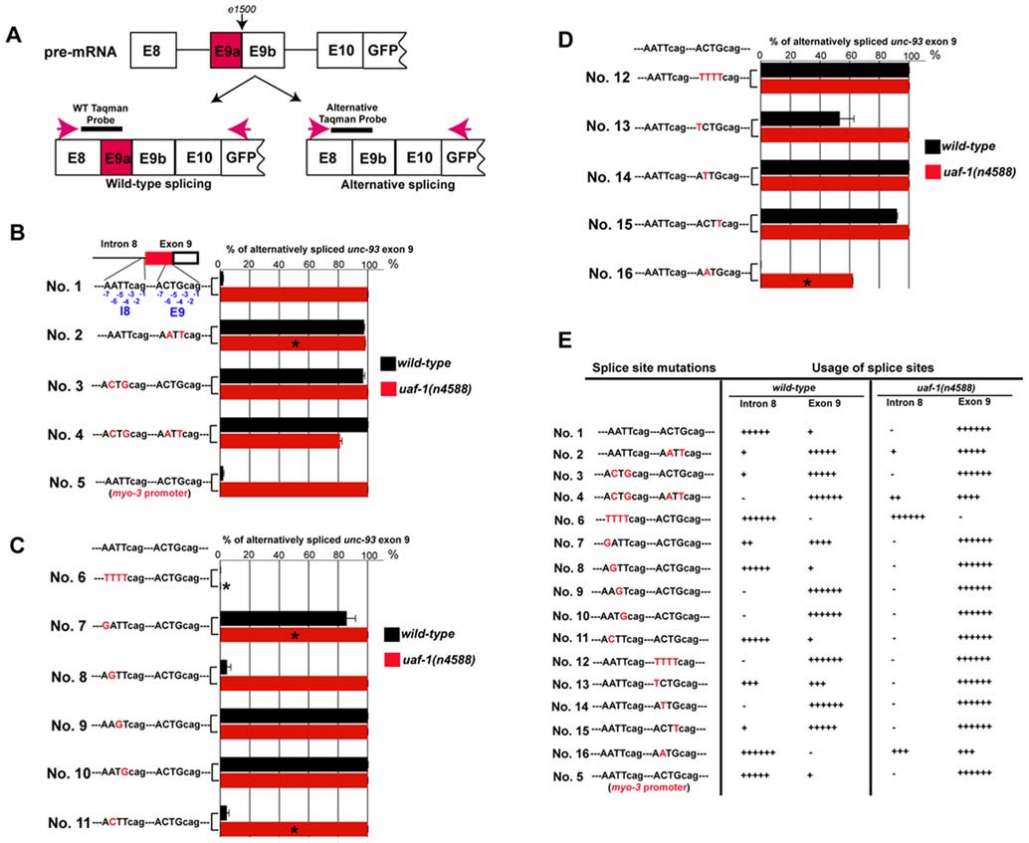
Nucleotide substitutions at the intron 8 endogenous 3′ splice site and the exon 9 cryptic 3′ splice site affect splicing at these different sites in wild-type and *uaf-1(n4588)* animals. (A) Exon structures of transgenes used for 3′ splice site nucleotide substitution analysis. Location of PCR primers (red arrows) and Taqman probes (dark short lines) are indicated. (B–D) List of 3′ splice site nucleotide substitutions and the proportion of alternatively spliced exon 9 expressed as a percentage of total spliced (wild-type and alternatively spliced products) *unc-93* exon 9 in wild-type and *uaf-1(n4588)* animals carrying the corresponding transgenes. Nucleotide bases altered are in red, and bases that remain the same as in the original 3′ splice site are in black. The designated positions (−7 to −1) of each base are indicated in transgene No. 1 of (B). For each transgene, two stable lines were established for both wild-type and *uaf-1(n4588)* animals, except in cases labeled with *, for which only one stable transgenic line was established. Each dataset represents the average value of duplicate measurements of each biological sample. Error bars: standard deviations. (E) Summary graph of the nucleotide substitution analysis shown in (B–D), indicating the % usage of each 3′ splice site in wild-type and *uaf-1(n4588)* animals carrying the corresponding transgenes. −, 0 to 1%; +, 1 to 10%; ++, 10 to 30%; +++, 30 to 70%; ++++, 70 to 90%; +++++, 90 to 99%; ++++++, 99 to 100%.

The alternative splicing between the intron 8 endogenous 3′ splice site (I8) and the exon 9 cryptic 3′ splice site (E9) in wild-type and *uaf-1* mutant animals allows an analysis of the effects of different nucleotides on the *in vivo* recognition of these alternatively spliced sites. We constructed a transgene that fuses the genomic sequence between exon 8 and exon 10 of *unc-93(e1500)* and the GFP reporter gene (Figure 6A) and placed the fusion transgene under the control of 2 kb of the promoter region of *unc-93*. We used a pair of PCR primers (Figure 6A, red arrows) that recognize *unc-93* exon 8 and the GFP sequences to specifically amplify transgene cDNAs in RT–PCR experiments. The Taqman probes shown in Figure 4A were used to quantify the wild-type and alternatively spliced isoforms (Figure 6A). Because I8 and E9 have the same nucleotides at positions −3 to −1 (CAG), our mutagenesis analysis focused on nucleotides −7 to −4 (Figure 6B), which are variable and are known to be critical for recognition and binding by the U2AF complex [29], [63]–[65]. We named each of 16 transgene constructs 1–16 (Figure 6B–6E).

We examined the splicing of a transgene (Figure 6B and 6E, No. 1) containing the same I8 and E9 as *unc-93(e1500)*. In wild-type animals the splicing mimics that of the endogenous *unc-93(e1500)*, with very little splicing at E9 (Figure 6B, 1.8%, compare to 1.3% in Figure 4B). Splicing of the same transgene (No. 1) in *uaf-1(n4588)* mutants occurred almost exclusively at E9 (>99%) (Figure 6B); endogenous splicing of *unc-93(e1500)* was qualitatively but not quantitatively similar, occurring mostly at E9 (68%) (Figure 4B). These results suggest that the splicing of mutated *unc-93* transgenes could provide important information concerning the *in vivo* recognition of 3′ splice sites.

We replaced E9 with the sequence of I8 (Figure 6B, No. 2). In both wild-type and *uaf-1(n4588)* animals, splicing occurred mostly at the new E9 (97% and 98%, respectively) (Figure 6B and 6E). When I8 was replaced with the sequence of E9 (Figure 6B, No. 3), splicing again occurred mostly at E9 in both wild-type and *uaf-1(n4588)* animals (97% and >99%, respectively) (Figure 6B and 6E). These results suggest that the sequence that surrounds the original E9 is preferred by the splicing machinery in both wild-type and *uaf-1(n4588)* animals when two identical 3′ splice sites are present. We next switched the positions of I8 and E9 (Figure 6B, No. 4). In the wild type most splicing (>99%) occurred at the new E9 (Figure 6B and 6E). Similarly, in *uaf-1(n4588)* animals, most splicing (80%) occurred at the new E9 (Figure 6B and 6E, No.4). However, that a significant amount of splicing (20%) occurred at the new I8 in *uaf-1(n4588)* animals (Figure 6B and 6E, No. 4) suggested that the mutant UAF-1 can efficiently recognize the original E9 sequence even at the I8 position, which is normally a less favorable position.

The pattern of alternative splicing in cell culture can depend on the promoter used [66]. *unc-93* is expressed in body-wall muscles [34],[37]. We tested whether a different muscle-specific promoter would alter the splicing pattern of transgene No. 1 by expressing the transgene under the control of a *myo-3* promoter [49] (Figure 6B and 6E, No. 5). We found almost identical splicing patterns of the transgene driven by the *myo-3* promoter and the *unc-93* promoter (Figure 6B and 6E, compare No. 1 and No. 5), suggesting that the alternative splicing of *unc-93(e1500)* involves a mechanism that is not promoter-specific.

We examined the effects of base substitutions at I8. Replacing I8 with the *C. elegans* consensus 3′ splice site TTTTcag [29], [63]–[65] caused splicing to occur exclusively (100%) at the new I8 in both wild-type and *uaf-1(n4588)* animals (Figure 6C and 6E, No. 6). To identify the nucleotides required for the recognition of I8 in wild-type animals, we substituted each base from −7 to −4 of I8 with a G (Figure 6C, No. 7 to No. 10). G is the least used nucleotide from −7 to −4 of identified 3′ splice sites [29],[64] and in previous studies substituting T with G at any of the four bases from −7 to −4 of the highly consensus TTTTcag site significantly compromised binding of the U2AF complex to this site [29]. A G substitution at −7 (No. 7), −5 (No. 9) and −4 (No. 10) of I8 all dramatically reduced splicing at the new I8 (to the level of 15%, 0%, 0%, respectively; Figure 6C and 6E) in wild-type animals, suggesting that these nucleotides are critical for the recognition by wild-type UAF-1. However, a G substitution at −6 (Figure 6C and 6E, No. 8) did not cause a significant change of splicing at the new I8 (which is 96% compared to 98% of No. 1), suggesting this nucleotide is not essential for recognition by wild-type UAF-1. We also substituted the A at −6 with a C to generate an I8 more similar to E9 (Figure 6C and 6E, No. 11). Splicing at this I8 (No. 11) was similar to that of transgenes No. 1 and No. 8 in wild-type animals, consistent with the notion that this base is not essential for the recognition by wild-type UAF-1. For all the transgenes with single-base substitutions of I8, splicing (Figure 6C and 6E, No. 7, 8, 9, 10 and 11) in *uaf-1(n4588)* animals is similar to that of transgene No.1 (Figure 6B and 6E), suggesting that none of the substitutions significantly increased the affinity of I8 for mutant UAF-1.

To test whether we could increase the recognition of E9, we replaced E9 with the highly conserved consensus 3′ splice site TTTTcag sequence [29], [63]–[65] (Figure 6D and 6E, No. 12). As expected, in both wild-type and *uaf-1(n4588)* animals, splicing occurred exclusively at the new E9 (100% and 100%, respectively). We next changed each of the non-T bases to T from −7 to −4 of E9 (Figure 6D and 6E, No. 13, 14 and 15). A T at −7, −6 or −4 increased splicing at E9 in wild-type animals (Figure 6D and 6E, No. 13, 14 and 15)(53%, 100% and 91% for positions −7, −6 and −4, respectively), suggesting these substitutions increased recognition of E9 by wild-type UAF-1. For all three of these transgenes, splicing in *uaf-1(n4588)* animals occurred exclusively at E9 (100% for all three) (Figure 6D and 6E, No. 13, 14, and 15), suggesting none of the T substitutions significantly reduced the recognition of E9 by mutant UAF-1.

We mutated the C at −6 of E9 to an A (Figure 6D and 6E, No. 16), generating a 3′ splice site with a T-to-G substitution at −4 of transgene No. 2 (Figure 6B, 6D, and 6E). Splicing of this transgene occurred exclusively at I8 (100%) in wild-type animals (Figure 6D and 6E, No. 16). In *uaf-1(n4588)* animals, splicing at the new E9 was reduced (to 62%, Figure 6D and 6E, No. 16) compared to that at E9 of transgene No. 2 (100%, Figure 6B and 6E, No. 2).

## Discussion

### Regulation of *unc-93* activity by *uaf-1* and *sfa-1* likely involves unknown genes and biological processes

The mechanism(s) of the suppression of the Unc phenotype caused by the *unc-93(e1500)* mutation by *uaf-1(n4588)* and *sfa-1(n4562)* remains to be determined. Four observations indicate that although *uaf-1(n4588)* causes alternative splicing of *unc-93(e1500)* exon 9, this alternative splicing is not the basis of the suppression. First, *unc-93(e1500)/unc-93(lf)* heterozygous animals are as Unc as *unc-93(e1500)* homozygous animals [36], indicating that reducing *unc-93* expression by 50% does not reduce the rubberband Unc phenotype. By contrast, *uaf-1(n4588 n5127)* reduced *unc-93(e1500)* expression by 25% (since there was 25% alternative splicing), and these animals were strongly suppressed. Also, in *uaf-1(n4588) unc-93(e1500)* animals, the *unc-93(e1500)* transcript was reduced by 68% (there was 68% alternative splicing), and these animals might have been expected to be slightly less Unc than *unc-93(e1500)/unc-93(lf)* animals but instead were strongly suppressed. Thus, the level of reduction of the *unc-93(e1500)* transcript does not correlate with the level of the suppression of the *unc-93(e1500)* Unc phenotype by *uaf-1(n4588 n5127)* and *uaf-1(n4588)*. Second, the strong rubberband Unc phenotype caused by overexpression of the *unc-93(e1500)*-specific cDNA in body-wall muscles was partially suppressed by *uaf-1(n4588)*, suggesting that *unc-93(e1500)* splicing is not needed for *uaf-1(n4588)*-mediated suppression. Third, *sfa-1(n4562)* suppressed *unc-93(e1500)* without affecting the splicing of *unc-93(e1500)* exon 9, and *sfa-1(n4562)* partially suppressed the rubberband Unc phenotype caused by overexpression of the *unc-93(e1500)* cDNA in the body-wall muscles. Again, suppression can occur without affecting *unc-93(e1500)* mRNA splicing. Similarly, we did not identify an alternatively spliced *sup-10* transcript in either *uaf-1(n4588)*; *sup-10(n983)* or *sfa-1(n4562)*; *sup-10(n983)* animals, suggesting that *sup-10(n983)* was suppressed by *uaf-1(n4588)* and *sfa-1(n4562)* by a mechanism other than alternative splicing of the *sup-10* transcript. Fourth, reducing the expression of *uaf-1* and *sfa-1* by RNAi suppressed the rubberband Unc phenotype of *unc-93(e1500)* but did not cause altered splicing of *unc-93(e1500)* exon 9. Based on these arguments, we propose that *uaf-1* and *sfa-1* mutations suppress *unc-93(e1500)* and *sup-10(n983)* by affecting the splicing of one or more unidentified genes required for the expression of the *unc-93(e1500)* and *sup-10(n983)* rubberband Unc phenotype. We cannot exclude the possibility that the alternative splicing of *unc-93(e1500)* contributed to the suppression of *unc-93(e1500)* by *uaf-1* mutations.

### *uaf-1* and *sfa-1* represent a new class of suppressors of the rubberband phenotype

The known suppressors of gf mutations of *unc-93*, *sup-9* and *sup-10* are of three classes. First, lf mutations in any of these three genes are recessive suppressors of the rubberband Unc phenotypes caused by gf mutations in any of these three genes, because the functions of all three genes are necessary for expression of the Unc phenotype [35],[36],[43]. Second, rare gf mutations of *sup-11* are strong dominant suppressors of *unc-93(e1500)* and *unc-93(n200)* and partial recessive suppressors of *sup-9(n1550)* and *sup-10(n983)* [54]. The mechanism of *sup-11* suppression is unknown. Third, lf mutations of *sup-18* are strong recessive suppressors of *sup-10(n983)* and weak recessive suppressors of *unc-93(e1500)*, *unc-93(n200)* and *sup-9(n1550)* [35]. The mechanism of *sup-18* suppression is also unknown. *uaf-1(n4588)* and *sfa-1(n4562)* define a new class of suppressors: they are recessive and allele-specific for *unc-93* gf mutations (*unc-93(e1500)* was suppressed, but *unc-93(n200)* was not) but not gene-specific (*sup-10(n983)* was also suppressed). Previous genetic and molecular studies from our laboratory led to the hypothesis that UNC-93, SUP-9 and SUP-10 form a protein complex in the body-wall muscles [34]–[37]. The identification of multiple suppressors of the rubberband Unc phenotype with distinct suppression patterns suggests that the presumptive UNC-93/SUP-9/SUP-10 complex could have multiple *in vivo* functions regulated in different ways. As mentioned above, we propose that mutations in *uaf-1* and *sfa-1* affect the splicing of one or more unknown genes required for *unc-93* and *sup-10* activity. This unknown gene might be required specifically for the expression of the rubberband Unc phenotype caused by *unc-93(e1500)* or *sup-10(n983)* but have a negligible role in the expression of the rubberband Unc phenotypes caused by *unc-93(n200)* or *sup-9(n1550)*. Similarly, *sup-11* and *sup-18* could affect functions of the UNC-93/SUP-9/SUP-10 complex distinct from that affected by *uaf-1* and *sfa-1*.

### *uaf-1* and *sfa-1* mutants provide new approaches for the analysis of the *in vivo* functions of the U2AF large subunit and SF1/BBP

The SF1/BBP and U2AF proteins are critical splicing factors that regulate splicing by binding the branch-point sequence and the 3′ splice sites [1],[2], respectively. Mutations that affect the U2AF subunits and SF1/BBP in the yeasts *Saccharomyces cerevisiae* and *Schizosaccharomyces pombe* and the fruit fly *Drosophila melanogaster* have significantly facilitated the understanding of the *in vivo* function and regulation of these splicing factors [17], [27], [67]–[70]. Studies of *S. cerevisiae* identified genetic and biochemical interactions between the U2AF large subunit and SF1/BBP [17],[27], and studies of *S. pombe* provided *in vivo* evidence that the U2AF subunits are required for splicing [68],[70]. In *Drosophila* null mutations of the U2AF large or small subunits cause lethality [67],[69], hindering genetic analysis of these splicing factors. Similarly, in *C. elegans*, reducing the expression of *uaf-1* or *sfa-1* by RNAi causes lethality [61],[62], suggesting that these genes are essential for animal survival. We identified mutations that affect *uaf-1* and *sfa-1* and allow the survival of animals in permissive conditions, such as at lower temperatures or when derived from heterozygous mothers. These mutations provide a valuable resource for analyzing the function and regulation of the U2AF large subunit and SF1/BBP genes *in vivo* in animals.

### Mutations in UAF-1 alter the *in vivo* recognition of a 3′ splice site

The recognition of 3′ splice sites is achieved by interactions between SF1/BBP and the U2AF large and small subunits, which together bind specific intronic sequences [1],[2]. However, it is not clear how these factors regulate the choice of the correct splice site when two or more potential 3′ splice sites are proximal *in vivo*. Distinguishing different 3′ splice sites is a critical aspect of alternative splicing.

The *unc-93(e1500)* missense mutation generates a new cryptic 3′ splice site (AG) within exon 9 (ACTGc**a**g). This site differs from the consensus 3′ splice site for *C. elegans* (TTTTcag) [29] and is more rarely used by *C. elegans* than is TTTTcag or the intron 8 endogenous 3′ splice site (AATTcag) (Table S4). Based on *in vitro* studies, this cryptic site should not be or be more weakly recognized by UAF-1 compared to TTTTcag and probably the intron 8 site AATTcag [29]. In a wild-type background, the choice between the wild-type 3′ splice site of *unc-93(e1500)* intron 8 and the cryptic non-consensus site in *unc-93(e1500)* exon 9 followed this prediction, as only 1.3% of the splicing events utilized this cryptic 3′ splice site. Strikingly, however, the *n4588* missense mutation in *uaf-1* shifted this specificity, causing splicing to occur mostly at the cryptic site, generating 68% of aberrantly spliced transcripts. This result suggests that UAF-1 might play an important role in determining the choice among alternative 3′ splice sites *in vivo* (see discussion below).

The *n4588* mutation did not cause an apparent change of UAF-1 protein level, and reducing UAF-1 using RNAi did not increase the relative amount of alternatively spliced exon 9, suggesting that *uaf-1(n4588)* might alter UAF-1 function. However, RNAi-treatment did not abolish the expression of UAF-1 (Figure S2), and we might have failed to detect an effect of UAF-1 on the splicing of *unc-93(e1500)* exon 9 because of residual UAF-1 protein in RNAi-treated animals. Thus, the altered splicing of *unc-93(e1500)* exon 9 in *uaf-1(n4588) unc-93(e1500)* mutants might reflect the consequence of the absence of UAF-1 activity. It is also possible that *uaf-1(n4588)* causes both a loss of function and an altered function of UAF-1, which cause the suppression of the rubberband Unc phenotype of the *unc-93(e1500)* animals and the altered splicing of *unc-93(e1500)* transcript, respectively.

The other 14 introns of the *unc-93* transcript appeared to be spliced similarly in wild-type and *uaf-1(n4588)* animals, suggesting that *uaf-1(n4588)* did not alter the recognition of most wild-type 3′ splice sites. We also found that *uaf-1(n4588)* did not suppress the Unc phenotype caused by the *unc-52(e669)* mutation, which can be suppressed by mutations in the splicing factor genes *smu-1* and *smu-2*. We conclude that the *uaf-1(n4588)* mutation does not affect all cases in which alternative splicing is possible.

We isolated four intragenic suppressors of the temperature-sensitive lethality caused by *uaf-1(n4588)*. Three of the suppressors (*n4588 n5120*, *n4588 n5125* and *n4588 n5127*) carried both the original *n4588* mutation and a second site mutation in *uaf-1*. These three new *uaf-1* mutations reduced the alternative splicing of *unc-93(e1500)* exon 9 to levels intermediate between those of *uaf-1(n4588)* and wild-type animals. This finding supports the hypothesis that UAF-1 is important in 3′ splice-site choice. The fourth intragenic suppressor, *n5123*, affected the same site as the original *n4588* mutation by generating a phenylalanine codon different from both the wild-type codon (threonine) and the codon generated by the *n4588* mutation (isoleucine). The *uaf-1(n5123)* allele behaves like the *uaf-1(+)* allele, suggesting that this mutation restored the normal specificity of UAF-1. The amino acids affected by these *uaf-1* mutations (*n4588*, *n5120*, *n5123*, *n5125* and *n5127*) are confined to a region between the U2AF small subunit-interacting domain [47] and the first RRM domain [26],[45] (Figure 5). We postulate that this region of UAF-1 defines a domain of UAF-1 important for 3′ splice-site selection.

### The sequences and positions of 3′ splice sites together define the efficiency of splicing events

In *C. elegans*, the first two nucleotides (−2 to −1) of 3′ splice sites are more highly conserved than nucleotides −7 to −3 [29],[64], which affect the binding of the U2AF factors [29]. We sought to identify the nucleotides that affect the recognition of I8 and E9 by UAF-1 *in vivo*.

First, we conclude that the location of a 3′ splice site affects its recognition. We found that the location of E9 was preferred to that of I8 by both wild-type and mutant UAF-1 when identical 3′ splice sites were present at the two locations (Figure 6B, No. 2 and 3). However, this positional effect was not absolute. When the high-affinity 3′ splice site TTTTcag was placed at either of the two locations, the site with the TTTTcag was preferred by both wild-type and mutant UAF-1 (Figure 6, No. 6 and No. 12). That splicing using I8 and E9 (both are likely weak 3′ splice sites, since there are fewer such sites in *C. elegans* introns than there are copies of the strong 3′ splice site sequence TTTTcag (Table S4)) was more affected by position than was splicing using the sequence TTTTcag suggests that weak 3′ splice sites might be preferably used for alternative splicing, and, strong 3′ splice sites such as TTTTcag might be generally used for constitutive splicing. If so, we might identify alternatively spliced genes by searching apparently weak 3′ splice sites and then performing RT–PCR analyses. That TTTTcag is strongly recognized by mutant UAF-1 is consistent with our finding that the *uaf-1(n4588)* mutation does not appear to affect the splicing of most other introns of *unc-93* (Figure 2B), which have a 3′ splice site identical or highly similar to TTTTcag (data not shown).

Second, we conclude that the nucleotides at −7, −5 and −4 were more important than the nucleotide at −6 for wild-type UAF-1 to recognize the sequence of I8 (Figure 6, No. 7 to No. 10). The nucleotides at −4 and −5 appear to be more important than that at −7. That the nucleotide at −4 is more important than the nucleotide at −6 also appears to be the case for splicing at E9 by wild-type UAF-1 (No. 15 and No. 16, compared to No. 2, Figure 6), which indicates that nucleotide substitution at −4 (No. 16) dramatically reduced splicing and nucleotide substitution at −6 (No. 15) had a minimal effect at E9 in wild-type animals.

Third, substituting individual non-T nucleotides with T in E9 improved its recognition by wild-type UAF-1. The original I8 (AATTcag) and E9 (ACTGcag) are both rare 3′ splice sites compared to TTTTcag, which is found in about 26% of the approximate 40000 introns analyzed, and is the most commonly used 3′ splice site in *C. elegans* (Table S4) [29],[64]. I8 appears more frequently in introns than does E9 (Table S4), suggesting that E9 has a lower affinity for the wild-type UAF-1 than does I8. That the wild-type UAF-1 rarely recognized E9 even in the more favored position (Figure 6, No. 1) is consistent with this notion. We found that substituting any E9 non-T nucleotide with T could increase the recognition of E9 in wild-type animals (Figure 6, No. 13 to 15), and a T substitution at −6 and −4 had a much stronger effect than one at −7.

Fourth, we conclude that the T180I(*n4588*) mutation caused UAF-1 to be more tolerant of a G nucleotide at −4 of E9. In transgenes No. 11 and No. 16, a G substitution at −4 of E9 dramatically reduced splicing at E9 in wild-type animals but did not or only moderately affected splicing at E9 in *uaf-1(n4588)* mutants (Figure 6). The splicing of transgenes No. 1, No. 4 and No. 5 is consistent with this observation, implying that a G at −4 is more tolerated in *uaf-1(n4588)* animals than in wild-type animals. Based on these observations, we propose that the G nucleotide at position −4 of E9 is critical for its recognition by the mutant UAF-1.

### *In vivo* functions of UAF-1 and SFA-1 probably remain to be discovered

*In vivo* studies have suggested functions for the U2AF large subunit beyond regulating pre-mRNA splicing. For example, *Drosophila* mutants with a temperature-sensitive U2AF large subunit are defective in the nucleus-to-cytoplasm export of intronless mRNAs at elevated temperatures [71], suggesting that lack of U2AF large subunit function can affect mRNA export in addition to pre-mRNA splicing. Studies of SF1/BBP suggest that this splicing factor might not be essential for splicing *in vitro* or *in vivo*. Biochemical depletion of SF1/BBP in extracts from HeLa cells [72] and *S. cerevisiae* [73] or genetic depletion of SF1/BBP in extracts from *S. cerevisiae* [73] did not significantly affect splicing *in vitro*. Reducing SF1/BBP expression by RNAi in HeLa cells does not affect the splicing of several endogenous genes and a reporter gene [74]. That *uaf-1(n4588)* and *sfa-1(n4562)* suppressed the Unc phenotype of *unc-93(e1500)* but had different effects on the splicing of *unc-93(e1500)* mRNA at the cryptic 3′ splice site suggests that *uaf-1* and *sfa-1* could have both distinct and shared *in vivo* functions in *C. elegans*. Specifically, the splicing of some genes might be affected similarly by *uaf-1* and *sfa-1*, with other genes differentially affected. Alternatively, it is possible that *uaf-1(n4588)* has a stronger effect on the splicing of *unc-93(e1500)* exon 9, while *sfa-1(n4562)* has a weaker effect not detected in the experiments we performed.

The lack of conditionally viable mutants of the U2AF large subunit and SF1/BBP has impeded the analysis of the *in vivo* functions of these splicing factors in animals. The mutations we isolated affecting these two splicing factors should allow novel approaches for *in vivo* analyses of RNA splicing and of the functions of the U2AF large subunit and SF1/BBP in *C. elegans*. The transgene splicing system we developed provides an *in vivo* reporter assay for understanding the role of UAF-1 and possibly other splicing factors in regulating alternative 3′ splice site recognition.

## Materials and Methods

### Strains

*C. elegans* strains were grown at 20°C as described [55], except where otherwise specified. N2 (Bristol) was the reference wild-type strain. CB4856 (Hawaii) was used for mapping mutations using single-nucleotide polymorphisms [75]. Mutations used in this study include: LGI: *sup-11(n403)* [54]. LGII: *sup-9(n1550, n2287)* [34],[43], *unc-52(e444, e669)* [55],[60]. LGIII: *vab-6(e697)* and *dpy-1(e1)* [55], *uaf-1*(*n4588*, *n5120*, *n5123*, *n5125*, *n5127*, *n5222*Δ) (this study), *unc-93(lr12, n200, n1912, e1500)* [36],[37],[44], *sup-18(n1014)* [35]. LGIV: *egl-23(n601sd)* [52], *dpy-4(e1166)* [55], *sfa-1*(*n4562*, *n5223*Δ) (this study). LGX: *twk-18(e1913sd)* [53], *unc-58(e665sd)* [51] and *sup-10(n983, n3564)* [34],[35]. The translocation *nT1* IV;V with the dominant *gfp* marker *qIs51* [76] was used to balance the *sfa-1* locus, and the translocation *sC1(s2023)[dpy-1(s2170)]* (A. Rose, D. Baillie and D. Riddle, the Genetic Toolkit project) was used to balance the *uaf-1*(*n5222*Δ) locus.

### Clonal screen to identify *unc-93(e1500)* suppressor genes essential for animal survival

Synchronized L4 *unc-93(e1500)* animals (P_0_) were mutagenized with EMS (ethyl methanesulfonate) as described [55]. F_1_ progeny from these animals were picked to single wells of 24-well culture plates with OP50 bacteria grown on NGM agar. F_2_ progeny were observed using a dissecting microscope to identify animals with improved locomotion. From ∼10,000 F_1_ clones screened, 100 independent suppressed strains were isolated. 97 of the isolates, including two weak recessive sterile suppressors and 95 recessive fertile suppressors, were kept as frozen stocks for possible later study. Three stronger suppressors that caused or were closely linked to mutations that caused sterility (*n4562*) or ts-lethality (*n4588* and *n4564*) were chosen for further analysis. The analysis of *n4564* is ongoing.

### Cloning of *n4588* and *n4562*

We mapped *n4588* to the left of *dpy-1* on LGIII based on the suppression of *unc-93(e1500)* using standard methods. As the suppressor activity and ts-lethality were very closely linked, *e.g.*, more than 500 *n4588 unc-93(e1500)/+ unc-93(e1500)* individuals failed to segregate Sup non-Let progeny, we then followed the phenotype of ts-lethality to further map *n4588*. We mapped *n4588* to the right of nucleotide 186577 on BE0003N10 (cosmid BE0003N10 sequences refer to nucleotides of accession no. AC092690) using 10 Vab recombinants recovered after crossing *vab-6(e697) n4588* hemaphrodites with males of the Hawaiian strain CB4856 [75] and to the left of nucleotide 13164 on Y92C3A (accession no. AC024874) using 37 Dpy recombinants recovered after crossing *n4588 dpy-1(e1)* hemaphrodites with males from the Hawaiian strain CB4856. We determined the coding sequences of four genes in this interval, *uaf-1*, *rab-18*, *kbp-4* and *par-2*, and identified a missense mutation in the third exon of the *uaf-1a* isoform.

As the suppressor activity and sterility of *n4562* were very closely linked, *e.g.*, over 500 *unc-93(e1500)*; *n4562/+* individuals failed to segregate Sup non-Ste progeny, we followed the sterility phenotype to map *n4562* to the right of *dpy-4* on LG IV using standard methods. We next mapped *n4562* to the right of nucleotide 37163 on Y43D4A (accession no. AL132846) using 234 Dpy recombinants recovered after crossing *dpy-4(e1166) n4562* with males of the Hawaiian strain CB4856. The sequences of coding exons of *sfa-1* and *uaf-2*, both located in this region, were determined, and a Cys458Opal (TGT-to-TGA) mutation was identified in *sfa-1*.

### Isolation of deletion alleles

Genomic DNA pools from EMS-mutagenized animals were screened for deletions using PCR as described [77]. Deletion mutant animals were isolated from frozen stocks and backcrossed to the wild type at least three times. *uaf-1*(*n5222*Δ) removes nucleotides 9786 to 11082 of YAC Y92C3B. *sfa-1*(*n5223*Δ) removes nucleotides 207818 to 208925 of YAC Y116A8C.

### Quantitative RT–PCR

Total RNA was prepared using Trizol according to the manufacture's instructions (Invitrogen), treated with RNase-Free DNase I (New England Biolabs) and followed by incubation at 75°C for 10 minutes to inactivate DNase I. First-strand cDNA was synthesized with random hexamer primers (New England Biolabs) using the Superscript II or III First-Strand Synthesis Kit (Invitrogen). Quantitative RT–PCR was performed using either a DNA Engine Opticon System (MJ Research) or a Mastercycler realplex system (Eppendorf). For the SYBR green-based assay (DNA Engine Opticon System), each 30 µl PCR reaction contained 1 to 10 ng RT template, 0.5 mM PCR primers and 15 µl 2× SYBR Green PCR Master Mix (Applied Biosystems). Three independent samples of synchronized wild-type (N2) and *uaf-1(n4588)* L1 animals were prepared, and levels of control genes (*rpl-26*, *gpd-2*, *act-1*) and tested genes (*myo-3*, *unc-93*, *sup-9*, *sup-10*, *sup-11*, *sup-18*) were quantified from each biological replicate. For the Taqman probe-based assay (Mastercycler realplex system), the probes (Figure 4A) were labeled at their 5′-ends with 6-carboxyfluorescein (FAM) and at their 3′-ends with Black Hole Quencher (BHQ-1) (Integrated DNA Technologies). Two independent samples of each genotype of animals of mixed stages were prepared, and levels of *rpl-26* and *unc-93* wild-type and alternatively spliced transcripts were quantified from each biological replicate. For RNAi-treated animals (see below), one sample for each assay was quantified. PCR primers and Taqman probes are listed in Table S5.

### Screen for suppressors of *uaf-1(n4588)*

Synchronized *uaf-1(n4588)* animals (P_0_) at the L4 larval stage grown at 15°C were mutagenized with EMS as described [55]. These P_0_ animals were allowed to grow to young adults at 15°C in a mixed population and bleached, and F_1_ progeny were synchronized at the early L1 stage by starvation in S medium [78]. The F_1_ animals were placed on 50 Petri plates (∼1000 animals/plate) with NGM agar seeded with OP50 and permitted to grow to young adults at 15°C and then moved to 25°C. After six days at 25°C, the animals were grown at 20°C for six days and examined each day for the presence of living F_2_ animals. About 50,000 F_1_ progeny from a mixture of more than 10,000 P_0_ animals were screened. From the screen, we recovered 13 surviving F_2_ animals from 13 different F_1_ plates. Six of the 13 suppressors were intragenic suppressors representing four different mutations: one was *n5120*, one was *n5123*, three were identical to *n5125* and one was *n5127*. It is possible that the three isolates containing the *n5125* mutation were derived from the same P_0_ animal, because all of the P_0_ animals were in a mixed population when bleached to release eggs. The other seven suppressors were extragenic mutations, *i.e.*, *n4588/+*; *sup/+* animals segregated *n4588*-like progeny. Some or all of the extragenic isolates could have been derived from the same P_0_ animal.

### RNA interference

Young adult animals (wild-type or *unc-93(e1500)*) were fed HT115(DE3) bacteria containing plasmids directing the expression of dsRNAs targeting either *uaf-1* or *sfa-1* on NGM plates with 1 mM IPTG and 0.1 mg/ml Ampicillin [79]. Surviving F_1_ progeny of the *unc-93(e1500)* animals (escapers) were examined for suppression of locomotion defects. Animals were washed from plates, rinsed three times with H_2_O, and resuspended in Trizol (Invitrogen) for preparation of total RNA or in 2× protein loading buffer (see Western blots, below) for SDS-PAGE analysis. We generated the DNA construct expressing dsRNA targeting *uaf-1* (see below). The bacterial strain expressing dsRNA targeting *sfa-1* was obtained from a whole-genome RNAi library [80], and the sequences of plasmids from single colonies of the strain were determined to confirm the presence of *sfa-1* coding sequences.

### Quantification of locomotion and the rubberband phenotype

L4 animals were picked 16–24 hrs before assaying and were grown at 20°C. Young adults were then individually picked to Petri plates containing NGM agar seeded with OP50, and bodybends were counted for 30 seconds using a dissecting microscope as described [81]. The rubberband phenotype was scored as described [43].

### Western blots

Animals were washed from plates with H_2_O, rinsed three times with H_2_O, resuspended in one volume of 2× SDS loading buffer (100 mM Tris.Cl (pH 6.8), 200 mM DTT, 4% SDS, 0.2% bromophenol blue, 20% glycerol), boiled for 5 minutes, and samples were then loaded onto 8% polyacrylamide gels containing SDS. Protein samples were transferred from polyacrylamide gels to Immobilon-P Transfer Membranes (Millipore). Primary and secondary antibody incubations were performed with 5% non-fat milk in TBST (25 mM Tris-HCl, PH 8.0; 125 mM NaCl; 0.1% Tween-20) at room temperature for one hour each. Signals were visualized using Chemiluminescence Reagent Plus (PerkinElmer Life Sciences) and X-ray film (BioMax XAR film, Kodak). Primary antibody was rabbit anti-UAF-1(1∶20000) [45]. Secondary antibody was HRP-conjugated goat anti-rabbit (1∶3000) (BioRad).

### Plasmids

To rescue the suppression of the Unc phenotype of *unc-93(e1500)* by *uaf-1(n4588)* or *sfa-1(n4562)*, *uaf-1* and *sfa-1* cDNAs were subcloned to vector pPD93.97 using *BamH*I and *Age*I restrictions sites. *uaf-1b* cDNA was amplified with PCR using *uaf-1a* cDNA as template and subcloned to pPD93.97 using *BamH*I and *Age*I restrictions sites. *uaf-1a* cDNA was subcloned to pPD49.83 (for heat-shock induced expression of *uaf-1a* cDNA) using *BamH*I and *Sac*I restriction sites. An *Xho*I/*Spe*I fragment of *uaf-1a* cDNA subcloned in a pGEM-TA easy vector (Promega) was subcloned to pPD129.36 (for the *uaf-1* RNAi construct) using *Xho*I and *Nhe*I sites. To test whether the truncated *unc-93*(Δ) cDNA caused by altered splicing of the *unc-93(e1500)* transcript in *uaf-1(n4588)* mutants encodes a functional UNC-93 protein, *unc-93* cDNA and *unc-93*(Δ) cDNA were subcloned to pPD93.97 using *BamH*I(blunt) and *Age*I sites. To test whether *uaf-1(n4588)* or *sfa-1(n4562)* mutations could suppress the Unc phenotype caused by ectopic expression of the *unc-93(e1500)* cDNA, *unc-93(e1500)* cDNA was subcloned to pPD93.97 using *BamH*I(blunt) and *Age*I sites. To examine the effect of nucleotide substitutions on the recognition of the intron 8 endogenous 3′ splice site and the exon 9 cryptic 3′ splice site, we fused the genomic sequence between exon 8 and exon 10 of *unc-93(e1500)* in-frame with the GFP gene of pPD93.97 using *BamH*I and *Age*I sites. We replaced the *myo-3* promoter of pPD93.97 with a 2 kb promoter of *unc-93* using *Pml*I and *BamH*I sites. Point mutations in *uaf-1*(stop codons), the *unc-93* (*e1500* mutation) or mutated transgenes were introduced using QuickChange II or III Site-Directed Mutagenesis Kit (Stratagene) with primers containing corresponding mutations. PCR was performed using Eppendorf Cyclers, and DNA products were resolved using agarose gels. DNA sequence determination was performed with an ABI Prism 3100 Genetic Analyzer. PCR primers are listed in Table S5.

### Transgene experiments

Germline transgene experiments were performed as described [82]. Transgene mixtures generally contained 20 µg/ml 1 kb DNA ladder (Invitrogen), 20 µg/ml *Arabidopsis* genomic DNA and 10 µg/ml of the transgene of interest. When the transgene did not cause the expression of a GFP fusion protein, 10 µg/ml pPD95.86-GFP plasmid (expressing GFP in body-wall muscles) or 5 µg/ml p*_myo-2_*dsRED (expressing RFP in pharynx) was added to the injection mixture as a visible fluorescence marker to identify animals carrying the transgene.

### Bioinformatics

We downloaded approximate 40,000 unique intronic sequences from WormMart (WormBase Release 195) and processed the sequences using BBEdit and MS Excel softwares. Identical 3′ splice sites (positions −7 to −1) were grouped and counted.

## Supporting Information

Figure S1 *uaf-1(n4588)* does not reduce the mRNA levels of genes that genetically interact with *unc-93*. Real-time RT-PCR analyses of mRNA levels of constitutively expressed genes (*act-1*, *gpd-2*), a body wall muscle specific gene (*myo-3*) and genes involved in the rubberband Unc phenotype (*unc-93*, *sup-9*, *sup-10*, *sup-18*, *sup-11*). The levels of endogenous *rpl-26* mRNA of each sample were quantified in parallel real-time RT-PCR experiments and used as loading controls. Each data set represents the average of duplicate experiments of three biological replicates of synchronized L1 animals. Error bars, standard errors.
(0.32 MB TIF)

Figure S2 *uaf-1* RNAi reduces UAF-1 protein level. (A) Animals were fed bacteria expressing control empty vector or dsRNA targeting *uaf-1*. A western blot was prepared using a UAF-1 polyclonal antibody [45] and total protein extracted from these RNAi-treated animals. *uaf-1(RNAi)* dramatically reduced the protein level of UAF-1. (B) Coomassie blue staining indicated that the loading of total protein was similar among the samples. (C) Quantification using NIH ImageJ software of UAF-1 levels in wild-type or *unc-93(e1500)* animals treated with either control empty vector or *uaf-1(RNAi)*. *uaf-1(RNAi)* treatment reduced UAF-1 levels in both wild-type and *unc-93(e1500)* animals (35% and 23% of the levels in animals treated with control empty vectors, respectively).
(0.30 MB TIF)

Figure S3 *uaf-1(n4588)* and *sfa-1(n4562)* do not cause obvious alternative splicing of the *sup-10* transcript. (A) Total RNAs from animals of the indicated genotypes were prepared and RT-PCR experiments were performed to amplify the full-length *sup-10* cDNA using PCR primers covering the 5′ start codon and the 3′ stop codon. No *sup-10* transcript with an abnormal size was detected for wild-type, *sup-10(n983)*, *uaf-1(n4588)* and *uaf-1(n4588)*; *sup-10(n983)* animals. DNA sequences of the *sup-10* RT-PCR bands from all the four genotypes were determined. No *sup-10* transcript with altered splicing was observed. (B) No alternatively spliced *sup-10* transcript was identified from wild-type, *sup-10(n983)*, *sfa-1(n4562)* and *sfa-1(n4562)*; *sup-10(n983)* animals using RT-PCR experiments and DNA sequence determination. Genotypes are indicated at the top.
(0.37 MB TIF)

Figure S4 *uaf-1(n4588)* and *sfa-1(n4562)* cause synthetic lethality. Anesthesized animals of the indicated genotypes were observed with Nomarski optics. For wild-type, *uaf-1(n4588)* and *sfa-1(n4562)*, animals were photographed 24 hours after the mid-L4 stage (based on vulval invagination [Herman, et al]). For *uaf-1(n4588)*; *sfa-1(n4562)* double mutants, animals were observed each day for up to ten days after hatching. No animals grew beyond the size of a wild-type animal of the L2 larval stage. Two escapers of the *uaf-1(n4588)*; *sfa-1(n4562)* genotype that survived embryonic lethality are shown. Scale bar: 200 µm. (Herman T, Hartwieg E, Horvitz HR (1999) sqv mutants of Caenorhabditis elegans are defective in vulval epithelial invagination. Proc Natl Acad Sci U S A 96: 968–973.)
(0.79 MB TIF)

Table S1 *uaf-1(n4588)* does not cause synthetic defects with loss-of-function mutations of *unc-93*, *sup-9* and *sup-10* and does not suppress other Unc mutants. To examine whether *uaf-1* functions redundantly with *unc-93*, *sup-9 or sup-10*, we compared double mutants containing *uaf-1(n4588)* and lf mutations of *unc-93*, *sup-9* or *sup-10* to *uaf-1(n4588)* single mutants for locomotion, growth, and gross morphology. No differences were observed. Double mutants with *uaf-1(n4588)* and gf mutations of *unc-58*, *egl-23* and *twk-18* were compared to single mutants carrying these gf mutations, and no visible suppression of the Unc phenotypes was observed. Double mutants containing *uaf-1(n4588)* and *unc-52(e444)* or *unc-52(e669)* mutations were compared to single mutants of either *unc-52(e444)* or *unc-52(e669)* mutations, and no visible suppression of the Unc phenotypes was observed. Pvl: protruding vulva. Ste: sterile. Dpy: dumpy. Egl: egg-laying defective.
(0.04 MB DOC)

Table S2 The alternatively spliced *unc-93* transcript does not encode a functional UNC-93 protein product. Transgenes driving the expression of the alternatively spliced *unc-93* cDNA (*unc-93* cDNA(Δ)) did not rescue the suppression of *sup-9(n1550)* by the *unc-93(lr12*Δ) mutation, while transgenes expressing a wild-type *unc-93* cDNA rescued the suppression.
(0.02 MB DOC)

Table S3 The alternatively spliced *unc-93* transcript likely does not encode a dominant-negative UNC-93 protein product. Tansgenes driving the expression of the *unc-93* cDNA(Δ) did not suppress the rubberband Unc phenotype of *unc-93(e1500)* animals, while transgenes expressing a wild type *unc-93* cDNA suppressed the Unc phenotype. As loss of function of *unc-93* results in phenotypically wild-type animals, the lack of suppression of *unc-93(e1500)* by the *unc-93*(Δ) transgenes suggests that the function of *unc-93* was not antagonized by *unc-93*(Δ), indicating that the *unc-93*(Δ) cDNA does not encode a dominant-negative UNC-93 protein.
(0.02 MB DOC)

Table S4 Sequences and distributions of the three 3′ splice sites we analyzed. Approximate 40,000 unique introns were analyzed, and the numbers and ratios of all types of 3′ splice sites were calculated. The list here includes the three sites we analyzed in our mutagenesis experiments shown in Figure 6. TTTTcag is the most commonly used 3′ splice site.
(0.03 MB DOC)

Table S5 List of PCR primers and Taqman probes.
(0.10 MB DOC)
