# Supplementary figures and images for: Mutations in the Caenorhabditis elegans U2AF Large Subunit UAF-1 Alter the Choice of a 3′ Splice Site In Vivo
Source: PLoS Genet. 2009 Nov 6;5(11):e1000708. doi: 10.1371/journal.pgen.1000708 (PMC2762039; doi:10.1371/journal.pgen.1000708)

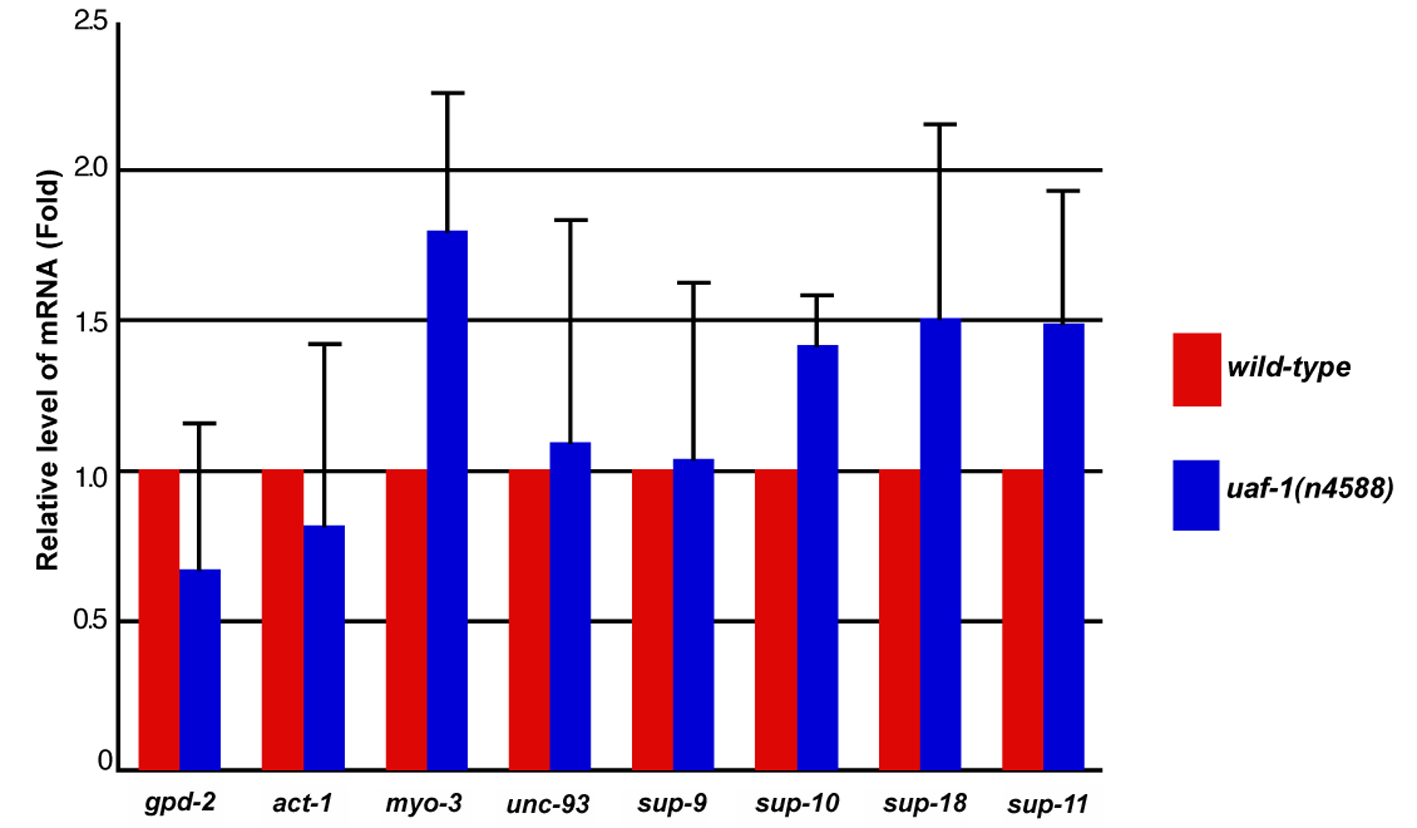

Supplement: Figure S1 — uaf-1(n4588) does not reduce the mRNA levels of genes that genetically interact with unc-93. Real-time RT-PCR analyses of mRNA levels of constitutively expressed genes (act-1, gpd-2), a body wall muscle specific gene (myo-3) and genes involved in the rubberband Unc phenotype (unc-93, sup-9, sup-10, sup-18, sup-11). The levels of endogenous rpl-26 mRNA of each sample were quantified in parallel real-time RT-PCR experiments and used as loading controls. Each data set represents the average of duplicate experiments of three biological replicates of synchronized L1 animals. Error bars, standard errors. (0.32 MB TIF) [file pgen.1000708.s001.tif]

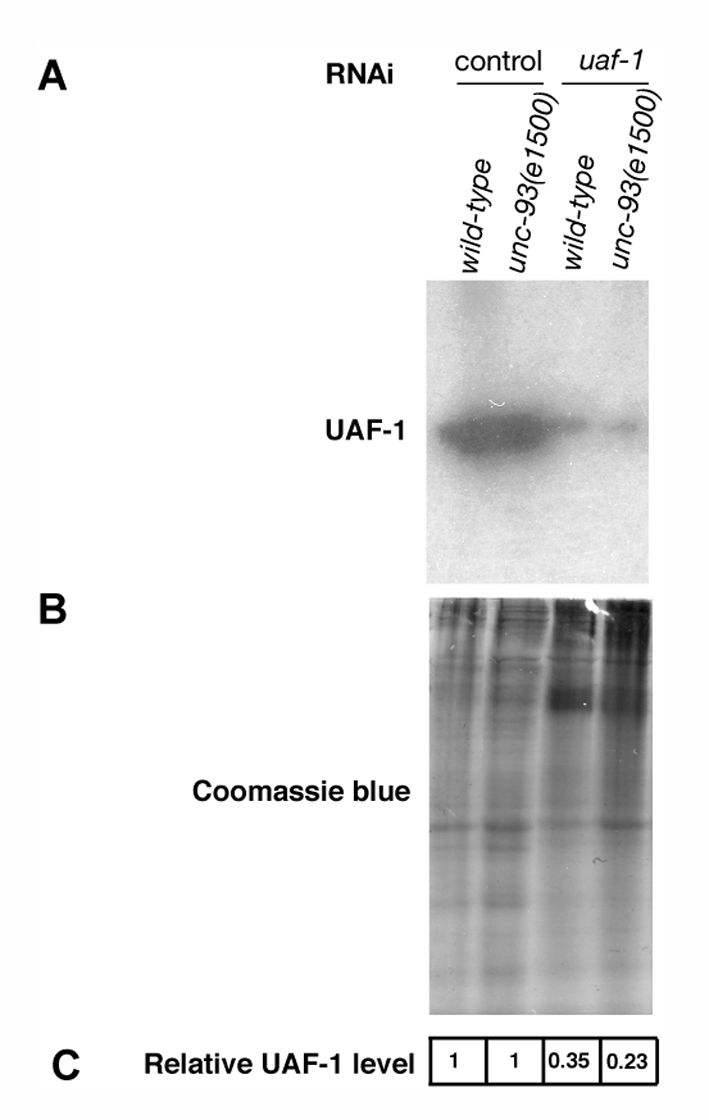

Supplement: Figure S2 — uaf-1 RNAi reduces UAF-1 protein level. (A) Animals were fed bacteria expressing control empty vector or dsRNA targeting uaf-1. A western blot was prepared using a UAF-1 polyclonal antibody [45] and total protein extracted from these RNAi-treated animals. uaf-1(RNAi) dramatically reduced the protein level of UAF-1. (B) Coomassie blue staining indicated that the loading of total protein was similar among the samples. (C) Quantification using NIH ImageJ software of UAF-1 levels in wild-type or unc-93(e1500) animals treated with either control empty vector or uaf-1(RNAi). uaf-1(RNAi) treatment reduced UAF-1 levels in both wild-type and unc-93(e1500) animals (35% and 23% of the levels in animals treated with control empty vectors, respectively). (0.30 MB TIF) [file pgen.1000708.s002.tif]

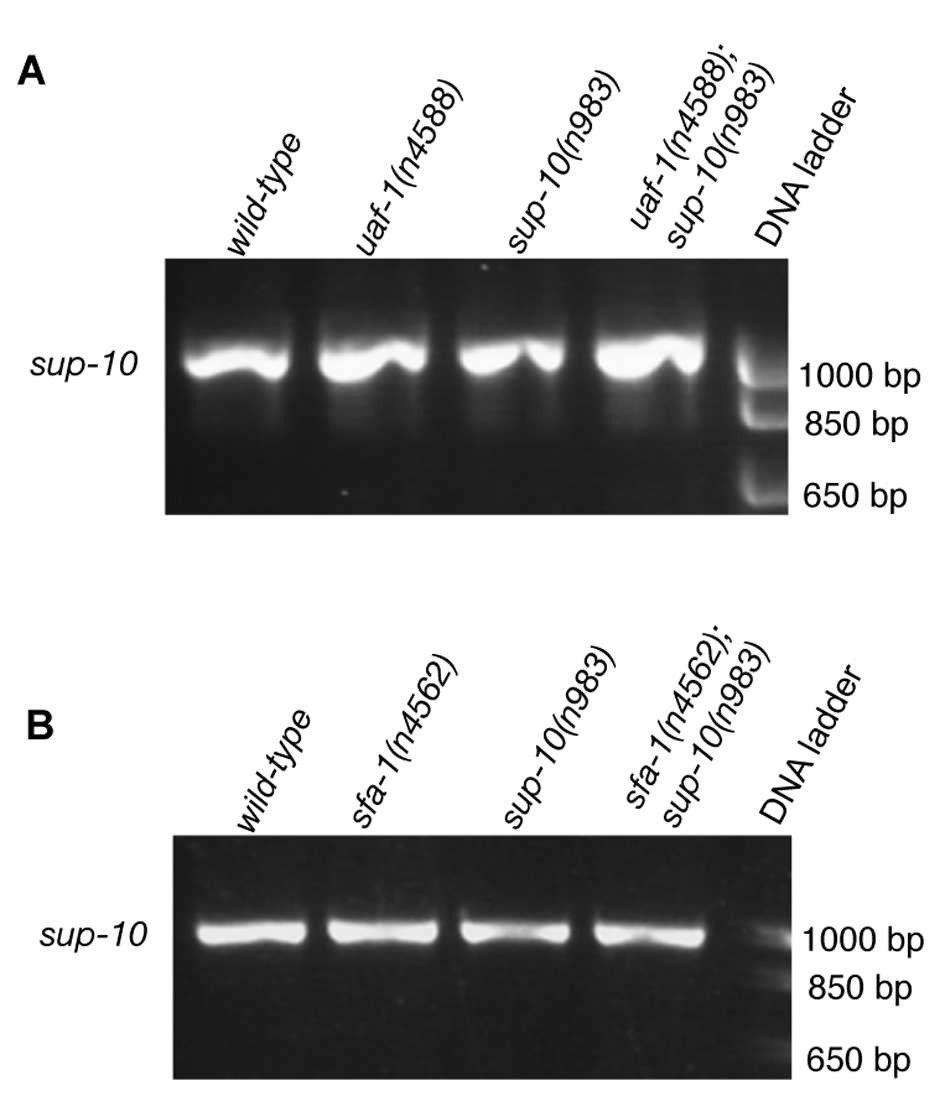

Supplement: Figure S3 — uaf-1(n4588) and sfa-1(n4562) do not cause obvious alternative splicing of the sup-10 transcript. (A) Total RNAs from animals of the indicated genotypes were prepared and RT-PCR experiments were performed to amplify the full-length sup-10 cDNA using PCR primers covering the 5′ start codon and the 3′ stop codon. No sup-10 transcript with an abnormal size was detected for wild-type, sup-10(n983), uaf-1(n4588) and uaf-1(n4588); sup-10(n983) animals. DNA sequences of the sup-10 RT-PCR bands from all the four genotypes were determined. No sup-10 transcript with altered splicing was observed. (B) No alternatively spliced sup-10 transcript was identified from wild-type, sup-10(n983), sfa-1(n4562) and sfa-1(n4562); sup-10(n983) animals using RT-PCR experiments and DNA sequence determination. Genotypes are indicated at the top. (0.37 MB TIF) [file pgen.1000708.s003.tif]

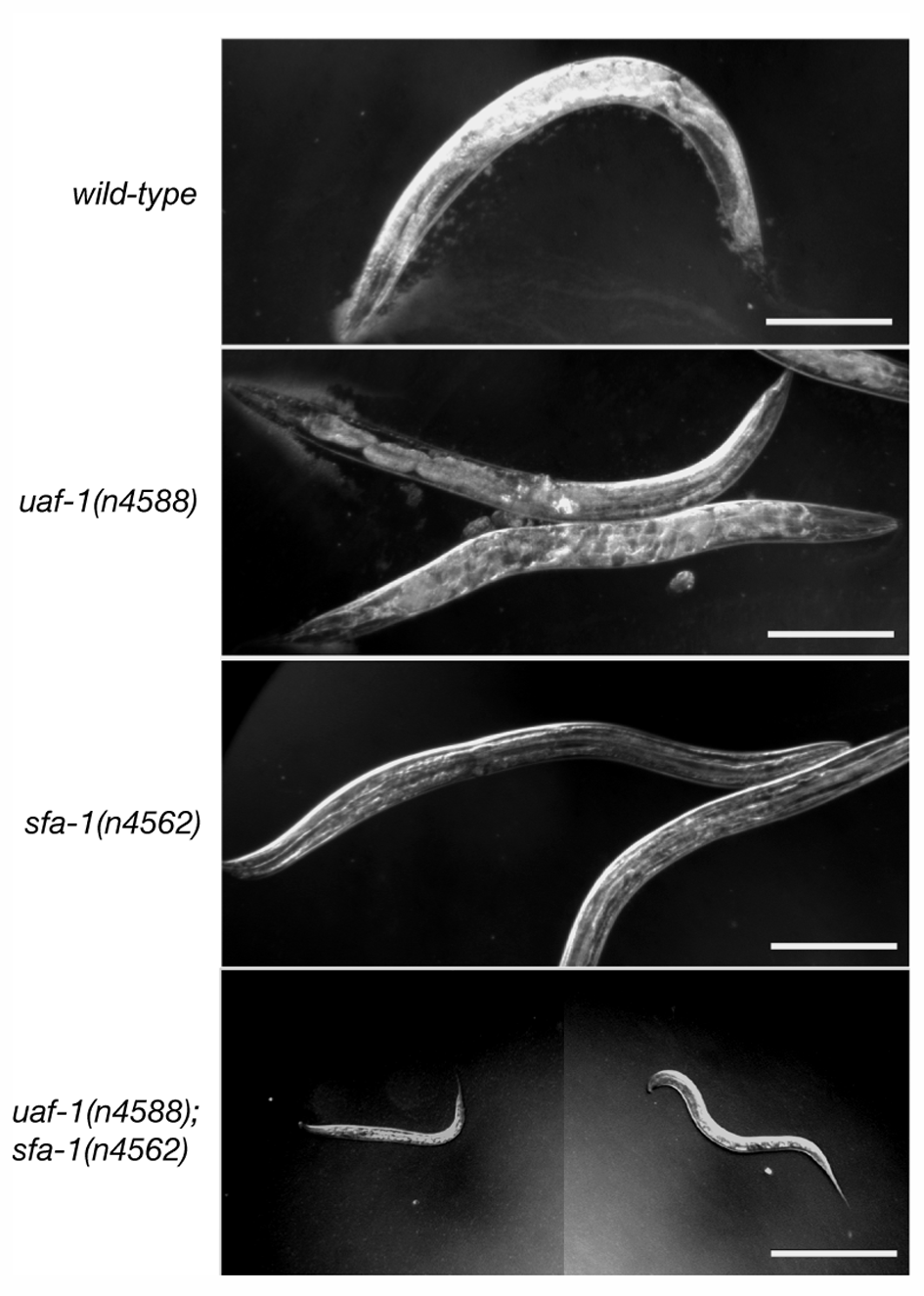

Supplement: Figure S4 — uaf-1(n4588) and sfa-1(n4562) cause synthetic lethality. Anesthesized animals of the indicated genotypes were observed with Nomarski optics. For wild-type, uaf-1(n4588) and sfa-1(n4562), animals were photographed 24 hours after the mid-L4 stage (based on vulval invagination [Herman, et al]). For uaf-1(n4588); sfa-1(n4562) double mutants, animals were observed each day for up to ten days after hatching. No animals grew beyond the size of a wild-type animal of the L2 larval stage. Two escapers of the uaf-1(n4588); sfa-1(n4562) genotype that survived embryonic lethality are shown. Scale bar: 200 µm. (Herman T, Hartwieg E, Horvitz HR (1999) sqv mutants of Caenorhabditis elegans are defective in vulval epithelial invagination. Proc Natl Acad Sci U S A 96: 968–973.) (0.79 MB TIF) [file pgen.1000708.s004.tif]
