# Supplementary material for: Mutations in the Caenorhabditis elegans U2AF Large Subunit UAF-1 Alter the Choice of a 3′ Splice Site In Vivo
Source: PLoS Genet. 2009 Nov 6;5(11):e1000708. doi: 10.1371/journal.pgen.1000708 (PMC2762039; doi:10.1371/journal.pgen.1000708)
Supplement: Table S1 — uaf-1(n4588) does not cause synthetic defects with loss-of-function mutations of unc-93, sup-9 and sup-10 and does not suppress other Unc mutants. To examine whether uaf-1 functions redundantly with unc-93, sup-9 or sup-10, we compared double mutants containing uaf-1(n4588) and lf mutations of unc-93, sup-9 or sup-10 to uaf-1(n4588) single mutants for locomotion, growth, and gross morphology. No differences were observed. Double mutants with uaf-1(n4588) and gf mutations of unc-58, egl-23 and twk-18 were compared to single mutants carrying these gf mutations, and no visible suppression of the Unc phenotypes was observed. Double mutants containing uaf-1(n4588) and unc-52(e444) or unc-52(e669) mutations were compared to single mutants of either unc-52(e444) or unc-52(e669) mutations, and no visible suppression of the Unc phenotypes was observed. Pvl: protruding vulva. Ste: sterile. Dpy: dumpy. Egl: egg-laying defective. (0.04 MB DOC) [file pgen.1000708.s005.doc]

| **Genotype** | **Phenotype (20^o^C)** |
| --- | --- |
| *uaf-1(n4588)* | Partial Pvl, partial Ste, weak Dpy |
| *uaf-1(n4588) unc-93(n1912)* | Partial Pvl, partial Ste, weak Dpy |
| *uaf-1(n4588); sup-10(n3564)* | Partial Pvl, partial Ste, weak Dpy |
| *sup-9(n2287); uaf-1(n4588)* | Partial Pvl, partial Ste, weak Dpy |
| *unc-58(e665sd)* | Shaker, rigidly paralyzed |
| *uaf-1(n4588); unc-58(e665sd)* | Shaker, rigidly paralyzed |
| *egl-23(n601sd)* | Egl, sluggish |
| *uaf-1(n4588); egl-23(n601sd)* | Egl, sluggish |
| *twk-18(e1913sd)/+* | Rubberband, paralyzed |
| *uaf-1(n4588); twk-18(e1913sd)/+* | Rubberband, paralyzed |
| *unc-52(e444)* | Adult paralyzed |
| *unc-52(e444); uaf-1(n4588)* | Adult paralyzed |
| *unc-52(e669)* | Adult paralyzed |
| *unc-52(e669); uaf-1(n4588)* | Adult paralyzed |
