# Supplementary material for: Mutations in the Caenorhabditis elegans U2AF Large Subunit UAF-1 Alter the Choice of a 3′ Splice Site In Vivo
Source: PLoS Genet. 2009 Nov 6;5(11):e1000708. doi: 10.1371/journal.pgen.1000708 (PMC2762039; doi:10.1371/journal.pgen.1000708)
Supplement: Table S2 — The alternatively spliced unc-93 transcript does not encode a functional UNC-93 protein product. Transgenes driving the expression of the alternatively spliced unc-93 cDNA (unc-93 cDNA(Δ)) did not rescue the suppression of sup-9(n1550) by the unc-93(lr12Δ) mutation, while transgenes expressing a wild-type unc-93 cDNA rescued the suppression. (0.02 MB DOC) [file pgen.1000708.s006.doc]

| **Genotype** | **Rescued lines/Total lines** |
| --- | --- |
| *sup-9(n1550); unc-93(lr12*Δ*); nEx[P_myo-3_unc-93 cDNA(*Δ*)::gfp]* | 0/15 |
| *sup-9(n1550); unc-93(lr12*Δ*); nEx[P_myo-3_unc-93 cDNA::gfp]* | 5/7 |
