# Supplementary material for: Mutations in the Caenorhabditis elegans U2AF Large Subunit UAF-1 Alter the Choice of a 3′ Splice Site In Vivo
Source: PLoS Genet. 2009 Nov 6;5(11):e1000708. doi: 10.1371/journal.pgen.1000708 (PMC2762039; doi:10.1371/journal.pgen.1000708)
Supplement: Table S3 — The alternatively spliced unc-93 transcript likely does not encode a dominant-negative UNC-93 protein product. Tansgenes driving the expression of the unc-93 cDNA(Δ) did not suppress the rubberband Unc phenotype of unc-93(e1500) animals, while transgenes expressing a wild type unc-93 cDNA suppressed the Unc phenotype. As loss of function of unc-93 results in phenotypically wild-type animals, the lack of suppression of unc-93(e1500) by the unc-93(Δ) transgenes suggests that the function of unc-93 was not antagonized by unc-93(Δ), indicating that the unc-93(Δ) cDNA does not encode a dominant-negative UNC-93 protein. (0.02 MB DOC) [file pgen.1000708.s007.doc]

| **Genotype** | **Suppressed Lines/Total Lines** |
| --- | --- |
| *unc-93(e1500); nEx[P_myo-3_unc-93 cDNA(*Δ*)::gfp]* | 0/5 |
| *unc-93(e1500); nEx[P_myo-3_unc-93 cDNA::gfp]* | 4/4 |
