# Supplementary material for: Mutations in the Caenorhabditis elegans U2AF Large Subunit UAF-1 Alter the Choice of a 3′ Splice Site In Vivo
Source: PLoS Genet. 2009 Nov 6;5(11):e1000708. doi: 10.1371/journal.pgen.1000708 (PMC2762039; doi:10.1371/journal.pgen.1000708)
Supplement: Table S4 — Sequences and distributions of the three 3′ splice sites we analyzed. Approximate 40,000 unique introns were analyzed, and the numbers and ratios of all types of 3′ splice sites were calculated. The list here includes the three sites we analyzed in our mutagenesis experiments shown in Figure 6. TTTTcag is the most commonly used 3′ splice site. (0.03 MB DOC) [file pgen.1000708.s008.doc]

|  | **Consensus** | **Intron 8** | **Exon 9** |
| --- | --- | --- | --- |
|  |  | | |
| **Sequence** | TTTTcag | AATTcag | ACTGcag |
| **Number** | 10770 | 632 | 22 |
| **Ratio** | 26% | 1.50% | 0.05% |
