# Supplementary material for: Mutations in the Caenorhabditis elegans U2AF Large Subunit UAF-1 Alter the Choice of a 3′ Splice Site In Vivo
Source: PLoS Genet. 2009 Nov 6;5(11):e1000708. doi: 10.1371/journal.pgen.1000708 (PMC2762039; doi:10.1371/journal.pgen.1000708)
Supplement: Table S5 — List of PCR primers and Taqman probes. (0.10 MB DOC) [file pgen.1000708.s009.doc]

| **Primers** | **Seqeunces** |
| --- | --- |
| *unc-93* Exon 8 P5 (5' red primer in Figure 2A): | GGAGCCAAATGCGCGTACATTACAG |
| *unc-93* Exon 9 P3 (3' red primer in Figure 2A): | GACCGCGTAGGGCTTGCGAATAGGAC |
| GFP Primer (3' red primer in Figure 6A): | CAACAAGAATTGGGACAACTCCAGTG |
| ***unc-93* RT-PCR primers (Figure2A, black arrows)** |  |
| exons 1-2 P5: | GTCAAGAGCAAGCCGTGTGGATTC |
| exons 1-2 P3: | TTGTGCCGTCTGGCTTCTTCTTC |
| exons 2-3 P5: | TCCTGATATTCCCTTGGAATC |
| exons 2-3 P3: | GCCTTCTGCTGTTCCCTGCCC |
| exons 3-4 P5: | CCAAAGTATTTTCCTTTCGAGAC |
| exons 3-4 P3: | GTCGTGGTCGAATGAGGTGCCAC |
| exons 4-5 P5: | CATGCATTCGGTAGATGATGAAG |
| exons 4-5 P3: | CATCATTTCTCGCTTCTTCTTCC |
| exons 5-6 P5: | GGAAGAAGAAGCGAGAAATGATG |
| exons 5-6 P3: | CAGGAAGAGAAAAGCCACGGAC |
| exons 6-7 P5: | CCAAAATCTTCAAACTTCCGTC |
| exons 6-7 P3: | GGAATGTTAATTTGCACCCGAG |
| exons 7-8 P5: | CTCGGGTGCAAATTAACATTCC |
| exons 7-8 P3: | CATGAAGCCGCGATTCCACAG |
| exons 8-9 P5: | GGAATTCGATATGCTAGCCTG |
| exons 8-9 P3: | GAGGATACCATATTTCCGACG |
| exons 9-10 P5: | ACACTGTCCTATTCGCAAGCC |
| exons 9-10 P3: | CAAGATTGCTCTCCGCCAACTC |
| exons 10-11 P5: | GAGTTGGCGGAGAGCAATCTTG |
| exons 10-11 P3: | CCAAAGCATTCAGGAACATGCTC |
| exons 11-12 P5: | GAGCATGTTCCTGAATGCTTTGG |
| exons 11-12 P3: | AACGGCACCAACAACATGAAC |
| exons 12-13 P5: | GACGATTTTCAATGGTTTGGAG |
| exons 12-13 P3: | GAGATTCCGAAGCAGGCCATCAC |
| exons 13-14 P5: | GCTTTTCGGTCGGATGCCTC |
| exons 13-14 P3: | CTACGTAGAATATTTGAGTGTCG |
| exons 14-15 P5: | TACAGTTTGCATTCACTAAATATCG |
| exons 14-15 P3: | GTAGCATGAAGAATGTTATCAGC |
| exons 15-16 P5: | GCTGATAACATTCTTCATGCTAC |
| exons 15-16 P3: | CTGGACATGACGTGTGAATTAAGTG |
| **Taqman PCR Probes (Figure 4):** |  |
| TaqmanProbeWT: | /56-FAM/A+CTA+CTGTTAT+TGTTAGATTTTTCG+GA+TATTTTTTCAT+GA /3BHQ_1/ |
| TaqmanProbeAlt: | /56-FAM/ACTG+TTAT+TGT+TAGACAAG+TCG+TC+GG/3BHQ_1/ |
|  | "+" represents Locked Nucleic Acids (LNA) |
| *rpl-26* Taqman PCR probe: | Ce02421921 (Purchased from Applied Biosystems) |
| **PCR primers for cDNAs of *unc-93, uaf-1* and *sfa-1*** |  |
| *unc-93* P5: | GAATTCATGAAATTTCAGAAAATGGGTGATTCAAAC |
| *unc-93* P3: | TTTTTCTACCGGTTCAAAATCTGAATTTCTATCATCCAACGG |
| *uaf-1a* P5: | GGATCCATGAGTGATCACCAGGATGGCATGAAACTTG |
| *uaf-1a* P3: | ACCGGTTGGAATTGACGATTGTGGTACTTGTCGACGTC |
| *uaf-1b* P5: | GGATCCATGCAACTCGGCGACAAGCAGCTCGTCGTTC |
| *sfa-1* P5: | GGATCCATGTCGAAAACTGGTGGAAACACGGAGCCAATG |
| *sfa-1* P3: | ACCGGTGGACTAGGCGGTGGCGGTGGAGCAGCGGCC |
| **PCR primers for *sup-10* full length cDNA (Figure S3)** |  |
| *sup-10* P5: | ATGCGATACGCGGTTTTTATATTTTTAATAGTG |
| *sup-10* P3: | CCCGGGGTCTTTATTGAATCCGGATCCATTG |
| **PCR primers for mRNA quantification (Figure S1)** |  |
| *sup-10* P5: | GGTGATGACATCAATCCATTAG |
| *sup-10* P3: | TCTACAATCGGATGCCAATACG |
| *sup-9* P5: | CGTCAGAGTTGGTGATCCTACTG |
| *sup-9* P3: | GCTTTTCATCTGGCAACTGATAGC |
| *unc-93* P5: | GTCAAGAGCAAGCCGTGTGGATTC |
| *unc-93* P3: | TTGTGCCGTCTGGCTTCTTCTTC |
| *sup-18* P5: | GAGGAGGCCTGAGAATGAAAG |
| *sup-18* P3: | GTTCGACCGGTTTTCTTTTGAG |
| *sup-11* P5: | TGAAGACTTGTGCATCACTTTGC |
| *sup-11* P3: | GCGCGTCAGTGTTATCCTTCTC |
| act-1 P5: | GCTGATCGTATGCAGAAGGAAATCAC |
| *act-1* P3: | AGGATAGATCCTCCGATCCAGAC |
| *myo-3* P5: | GCAAACTTAAGATCTTCAAGAGAC |
| *myo-3* P3: | CAGCTTGTTCAAATTGAGCAGTG |
| *gpd-2* P5: | CACCACCATCGAGAAGGCCAAC |
| *gpd-2* P3: | GGTTGACTCCGACGACGAACATTG |
| *rpl-26* P5*:* | ACTTCAACGCTCCATCCCAC |
| *rpl-26* P3: | AACGACGACCTCATCATCGG |
